# The *Arabidopsis* KINβγ Subunit of the SnRK1 Complex Regulates Pollen Hydration on the Stigma by Mediating the Level of Reactive Oxygen Species in Pollen

**DOI:** 10.1371/journal.pgen.1006228

**Published:** 2016-07-29

**Authors:** Xin-Qi Gao, Chang Zhen Liu, Dan Dan Li, Ting Ting Zhao, Fei Li, Xiao Na Jia, Xin-Ying Zhao, Xian Sheng Zhang

**Affiliations:** State Key Laboratory of Crop Biology, College of Life Sciences, Shandong Agricultural University, Tai’an, China; University of Massachusetts at Amherst, UNITED STATES

## Abstract

Pollen–stigma interactions are essential for pollen germination. The highly regulated process of pollen germination includes pollen adhesion, hydration, and germination on the stigma. However, the internal signaling of pollen that regulates pollen–stigma interactions is poorly understood. KINβγ is a plant-specific subunit of the SNF1-related protein kinase 1 complex which plays important roles in the regulation of plant development. Here, we showed that KINβγ was a cytoplasm- and nucleus-localized protein in the vegetative cells of pollen grains in *Arabidopsis*. The pollen of the *Arabidopsis kinβγ* mutant could not germinate on stigma, although it germinated normally *in vitro*. Further analysis revealed the hydration of *kinβγ* mutant pollen on the stigma was compromised. However, adding water to the stigma promoted the germination of the mutant pollen *in vivo*, suggesting that the compromised hydration of the mutant pollen led to its defective germination. In *kinβγ* mutant pollen, the structure of the mitochondria and peroxisomes was destroyed, and their numbers were significantly reduced compared with those in the wild type. Furthermore, we found that the *kinβγ* mutant exhibited reduced levels of reactive oxygen species (ROS) in pollen. The addition of H_2_O_2_ *in vitro* partially compensated for the reduced water absorption of the mutant pollen, and reducing ROS levels in pollen by overexpressing *Arabidopsis CATALASE 3* resulted in compromised hydration of pollen on the stigma. These results indicate that *Arabidopsis* KINβγ is critical for the regulation of ROS levels by mediating the biogenesis of mitochondria and peroxisomes in pollen, which is required for pollen–stigma interactions during pollination.

## Author Summary

After landing on the stigma, pollen grains germinate and create pollen tubes following adhesion and hydration processes, during which pollen–stigma interactions determine whether the pollen grains can germinate on the stigma. In recent years, the interaction mechanisms between the pollen and stigma have been studied extensively at the cellular and molecular level in self-incompatibility systems. However, few studies have focused on pollen–stigma interactions during self-compatible pollination. *Arabidopsis thaliana* provides an excellent system to study the interaction mechanisms between the pollen and stigma during self-compatible pollination. KINβγ is a plant-specific subunit of the SNF1-related protein kinase 1 complex. In this study, we identified an *Arabidopsis kinβγ* mutant showing defective pollen germination on the surface of the stigma but not on the culture medium, which resulted from the compromised hydration of pollen on the stigma. Further analysis revealed that the biogenesis of mitochondria and peroxisomes was impaired in this mutant, which reduced the levels of reactive oxygen species (ROS) in pollen. Application of H_2_O_2_ recovered the capability of pollen to undergo hydration *in vitro*. These results suggest that ROS signaling is involved in the regulation of pollen–stigma interactions during pollination. This study provides new insights into the mechanism underlying pollen–stigma interactions in self-compatible plant species.

## Introduction

In flowering plants, pollen tube growth provides a route for the delivery of nonmotile sperm to the female gamete. The growth of the pollen tube begins during the germination of the pollen grain after it lands on a receptive stigma via pollination. Pollen–stigma interactions are crucial for determining the germination of compatible pollen on the stigma [1, 2]. During the interactions between the pollen and stigma, a series of events occur in succession, including pollen adhesion onto the stigma, pollen hydration and germination, and the polarized growth of pollen tubes across stigmatic tissue. In recent years, the interactions between the pollen and stigma have been studied in a number of plant species [3, 4]. However, determining the mechanisms underlying these interactions remains a major challenge.

The stigmatic exudates at the stigma surface of wet stigma species provide the basis for pollen capture, adhesion, and hydration, which are considered passive, unregulated processes in these plant species. By contrast, there are no stigmatic exudates on the stigma surface in dry stigma species, such as *Arabidopsis*, maize, and *Brassica*. The pollen wall, which is thought to be important for pollen adhesion, hydration, and germination at the stigma surface during pollination [2], is involved in the formation of the pollen–stigma interface and promotes pollen adhesion and water flow from the stigma to the pollen grain. For example, the exine and exinic outer layer of pollen function in the initial adhesion of pollen to dry stigmas [5, 6]. Following exine-mediated adhesion, pollen coat (“foot”) formation between the pollen wall and stigma surface might further contribute to adhesion [1]. However, studies of the *Arabidopsis eceriferum6* (*cer6*) mutant revealed that the pollen coat is not essential for initial adhesion, but it may contribute to the magnitude of adhesion after hydration [6, 7]. Some studies suggest that the pollen coat is critical for pollen hydration and germination on the stigma. For example, the pollenkitt of sunflower pollen mediates pollen adhesion on the hydrophilic surface in a humidity-dependent manner [8]. *Arabidopsis cer* mutants (e.g., *cer1*, *cer3*, *cer6*) have defective pollen coats, which results from the lack of a class of long-chain lipids; these mutants demonstrate defective pollen hydration at wild-type stigma and cannot give rise to pollen tubes, thereby leading to male sterility [9].

Various proteins have been identified in the pollen coat and apoplast of *Arabidopsis* and maize [10–12], some of which are important for pollen–stigma interactions during pollination. For example, the oleosin-domain protein GRP17 and the lipase EXL4 are *Arabidopsis* pollen coat proteins that function in pollen hydration on the stigma [13, 14]. In maize, xylanase released from the pollen coat after pollination facilitates pollen tube penetration into the silk via enzymatic xylan hydrolysis [15]. These findings demonstrate that the molecules located at the surface of the pollen grain are critical for the interactions between pollen and the stigma during pollination; by contrast, there is little evidence supporting the role of signaling from the interior of pollen grains in self-compatible pollination. Recently, an *Arabidopsis dayu* mutant was identified, which has impaired pollen germination *in vivo* and reduced jasmonic acid levels in pollen [16]. DAYU/ABERRANT PEROXISOME MORPHOLOGY9 is a regulator of peroxisome biogenesis in pollen and is expressed in pollen vegetative cells, indicating that jasmonic acid signaling from the interior of pollen grains regulates pollen germination on the stigma in *Arabidopsis*.

The yeast sucrose nonfermenting 1 (SNF1)/mammal AMP-activated kinase (AMPK)/plant Snf1-related kinase1 (SnRK1)-related protein kinase complex belongs to a family of highly conserved heterotrimeric serine/threonine kinase complexes. This complex can be activated when ATP production decreases relative to increases in AMP or ADP levels in cells that are involved in cellular responses to various nutritional and environmental stresses [17–19]. The SNF1/AMPK/SnRK1 complex is composed of catalytic α subunits and two regulatory noncatalytic subunits: β and γ [20]. The β subunit functions in determining the subcellular localization of the complex and recognition between the kinase and its targets, acting as a scaffold to keep the α and γ subunits together; the γ subunit is involved in the regulation of SNF1/AMPK/SnRK1 kinase activity. The α subunit of *Arabidopsis* SnRK1 complex, is encoded by KIN10, KIN11, and KIN12, but only the expression of KIN10 and KIN11 has been detected in *Arabidopsis* [21, 22]. Recently, *Arabidopsis* SnRK1 was identified as an atypical member of the SNF1/AMPK/SnRK1 family due to its insensitivity to AMP and ADP, resistance to T-loop dephosphorylation, and inability to bind to starch or similar oligo/polysaccharides [23]. Genetic analyses revealed that SnRK1 plays roles in the regulation of seedling, embryo, pollen, and lateral organ development and is also involved in sugar, stress, and hormonal signaling [21, 24–29]. Three classes of putative γ subunits of the SnRK1 complex, i.e., KINγ, KINβγ/HOMOLOG OF YEAST SUCROSE NONFERMENTING 4 (SNF4), and PV42-type proteins, have been identified in plants [30, 31]. *Arabidopsis* AtPV42a and AtPV42b belong to the PV42 class of γ subunits. Artificial microRNA (amiRNA)-mediated knockdown mutants of both *atpv42a* and *atpv42b* exhibit defects in late stamen development and pollen tube growth to the female gametophyte [31]. KINβγ is an atypical γ subunit since it contains a glycogen-binding domain/kinase interaction sequence (GBD/KIS) in its N-terminal region that is normally found in canonical β subunits besides four cystathionine-β-synthase (CBS) domains in its C-terminal region and a pre-CBS domain located between the four CBSs and GBD domains [32]. *Arabidopsis* KINβγ is a functional homolog of the yeast γ subunit SNF4 and is assembled into a plant-specific SnRK1 complex by interacting with both the α and β subunits [23, 33–36]. As a unique regulatory subunit of the SnRK1 complex, KINβγ might have some plant-specific functions. KINβγ can form an active complex with the α-catalytic KIN10 and KIN11 subunits [37] and may be involved in plant–pathogen interactions by interacting with two leucine-rich repeat proteins [34]. Consistent with the lethal phenotypes of the *kin10*,*11* double mutant in *Arabidopsis*, no homozygous *kinβγ* knockout mutant could be isolated [21, 32], thereby indicating that KINβγ is critical to plant development.

In this study, we isolated heterozygous *kinβγ* knockout mutants in *Arabidopsis* and found that the pollen of these mutants exhibited abnormal biogenesis of mitochondria and peroxisomes and reduced reactive oxygen species (ROS) levels. Pollen grains of the *kinβγ* mutants germinated *in vitro* but not on the surface of the stigma, which resulted from the reduced ROS levels in the mutant pollen. This study provides new insights into the mechanism underlying the interactions between the pollen and the stigma.

## Results

### *Arabidopsis kinβγ* mutant exhibits defective pollen

**Fig 1:**
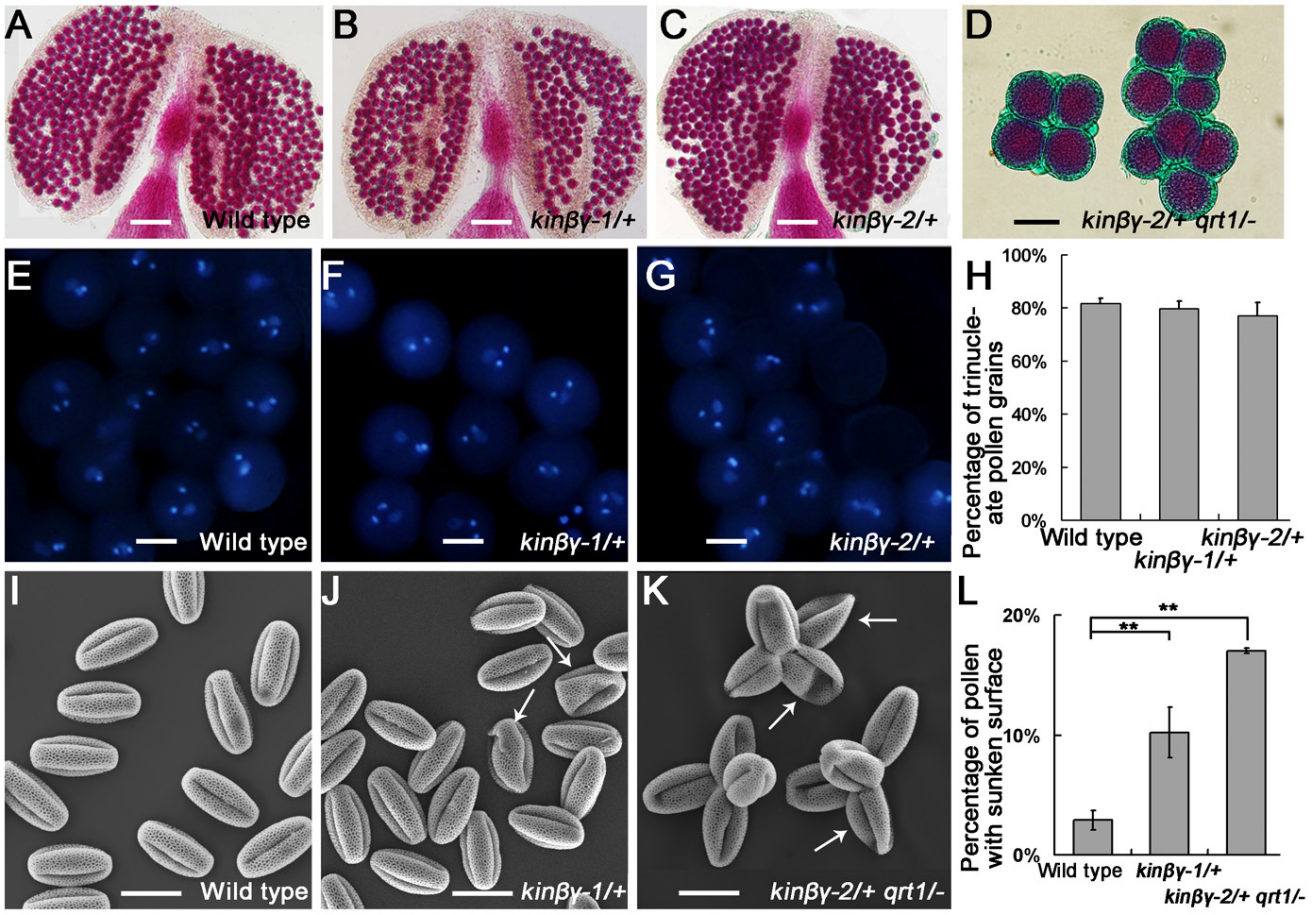
Viability and morphology of mature *kinβγ/+* pollen grains. (A–D) Alexander staining revealing the viability of mature pollen of the wild type (A), *kinβγ-1/+* (B), *kinβγ-2/+* (C), and *kinβγ-2/+ qrt1/-* (D). (E–G) DAPI labeling of the nuclei in mature pollen grains of the wild type (E), *kinβγ-1/+* (F), and *kinβγ-2/+* (G). (H) Statistical analysis of the trinucleate pollen grains of the wild type (E), *kinβγ-1/+* (F), and *kinβγ-2/+* (G). (I–K) SEM analysis of the morphology of mature pollen grains of the wild type (I), *kinβγ-1/+* (J), and *kinβγ-2/+ qrt1/-* (K). Arrows indicate the pollen grains with sunken surfaces. (L) Statistical analysis of pollen grains with sunken surfaces. Data were collected from three independent experiments. Asterisks indicate significant difference (Student’s *t*-test, P<0.01). Bars, 100 μm in (A–C) and 20 μm in (D–G) and (I–K).

In a previous study, homozygous *kinβγ* knockout mutant plants could not be identified from the progenies of *kinβγ* mutants in *Arabidopsis* [32]. To analyze the reason for the lack of *kinβγ* homozygous mutants, we obtained two *KINβγ* T-DNA insertion mutant lines, GK-346E09 and SALK_074210, from the European Arabidopsis Stock Centre and the Arabidopsis Biological Resource Center, respectively. Although no homozygous T-DNA insertion mutant plants were identified from the progenies of these mutant plants according to PCR-based genotype identification, two heterozygous mutant progenies from GK-346E09 and SALK_074210 were identified and designated as *kinβγ-1/+* and *kinβγ-2/+*, respectively (S1 Fig). Segregation analysis of the selfed progenies of these two mutants revealed that the ratios of wild-type/heterozygous plants were 1:1 (S1 Table), indicating that the T-DNA insertions in *KINβγ* led to gametophytic defects. Analysis of reciprocal crosses between the wild-type plants and the heterozygous mutants showed that there were significant decreases in the transmission of the mutant allele via the male gametophytes (S2 Table). The *Arabidopsis quartet1* (*qrt1*) mutant contains pollen tetrads instead of separate pollen grains, which is convenient for the identification of pollen phenotypes resulted from a gametophytic mutation [38]. To make a comparison of the pollen grains between the wild type and *kinβγ* mutant in the following analysis, we introduced *qrt1* into *kinβγ/+* mutant plants. The viability and nuclei of mature pollen were examined in the heterozygous *kinβγ-1*/+ and *kinβγ-2*/+ mutants using Alexander staining and 4',6-diamidino-2-phenylindole (DAPI)-labeling procedures, respectively. All of the mature pollen grains appeared red after Alexander staining, and most of them harbored three nuclei (Fig 1A–1H). Next, we observed the morphology of the mature pollen grains by scanning electron microscopy (SEM). There were no detectable differences in pollen size or sculpturing patterns in the exine between the mutants and wild type, but approximately 10.2% and 17.0% of grains in *kinβγ-1*/+ and *kinβγ-2*/+ *qrt1*/- had sunken surfaces, respectively. By contrast, only 2.9% of grains with sunken surfaces were identified in the wild type (Fig 1I–1L).

To confirm that the *kinβγ* mutation led to pollen defects, we performed a complementary analysis using the *SNF4-YFP* transgenic line, in which the genomic sequence of *KINβγ* (including the promoter and coding sequences) was fused with the *yellow fluorescent protein* (*YFP*) sequence and transformed into wild-type *Arabidopsis* [36]. We obtained *SNF4-YFP*/+ *kinβγ-1/+* plants by crossing the *kinβγ-1/+* plant with the *SNF4-YFP* transgenic plant. We determined the ratios of plants with and without T-DNA insertions (T-DNA^+^ and T-DNA^-^, respectively) in the offspring plants of both *kinβγ-1/+* and *SNF4-YFP*/+ *kinβγ-1/+*. The T-DNA^+^ to T-DNA^-^ ratio was 0.81:1 (93:115) in the offspring of the *kinβγ-1/+* plant, whereas it was 2.70:1 (165:61) in the offspring of the *SNF4-YFP*/+ *kinβγ-1/+* plant. Next, we observed the pollen morphology of *SNF4-YFP*/+ *kinβγ-1/-* and wild-type plants. The majority of pollen grains exhibited the normal morphology of the wild type (S2 Fig). The percentage of pollen grains with a sunken surface decreased to 5.2% in *SNF4-YFP*/+ *kinβγ-1/-*; by contrast, the percentage was approximately 10.2% in *kinβγ-1*/+ pollen grains. These results confirm that the defective phenotype of *kinβγ/+* pollen resulted from mutation of the *KINβγ* gene.

### Localization of KINβγ in pollen grains and pollen tubes

**Fig 2:**
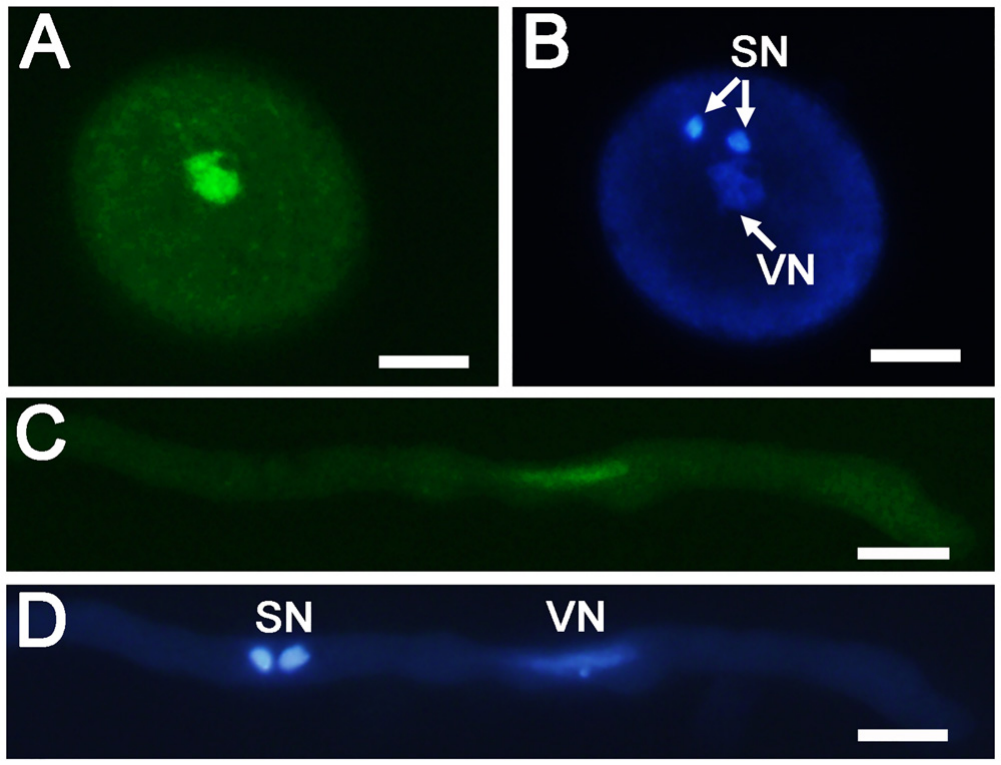
Localization of KINβγ in pollen and pollen tube. (A) and (B) Localization of KINβγ in the mature pollen grain expressing *SNF4-YFP*. (B) The pollen grain in (A) labeled with DAPI to visualize the nucleus. (C) and (D) Localization of KINβγ in pollen tube expressing *SNF4-YFP*. (D) The pollen tube in (C) labeled with DAPI to visualize the nucleus. SN, sperm nuclei; VN, vegetative nucleus. Bars, 10 μm

*Arabidopsis* KINβγ is a pollen-expressed protein [36]. To obtain detailed information about *Arabidopsis* KINβγ expression and its localization in pollen grains and pollen tubes, we observed YFP signals in the pollen grains and pollen tubes of the *SNF4-YFP* transgenic line. In mature pollen grains and pollen tubes, diffuse SNF4-YFP signals were distributed in the cytoplasm and concentrated signals were located in a nucleus-like structure (Fig 2A and 2C), which is consistent with the previous observation [36]. To confirm the localization of KINβγ in pollen grains and pollen tubes, we stained these structures with DAPI and found that KINβγ was mainly localized to the vegetative nuclei, in addition to the cytoplasm of vegetative cells, in pollen grain and pollen tube (Fig 2A–2D).

### Biogenesis and ultrastructure of mitochondria and peroxisomes are impaired in *kinβγ* mutant pollen

**Fig 3:**
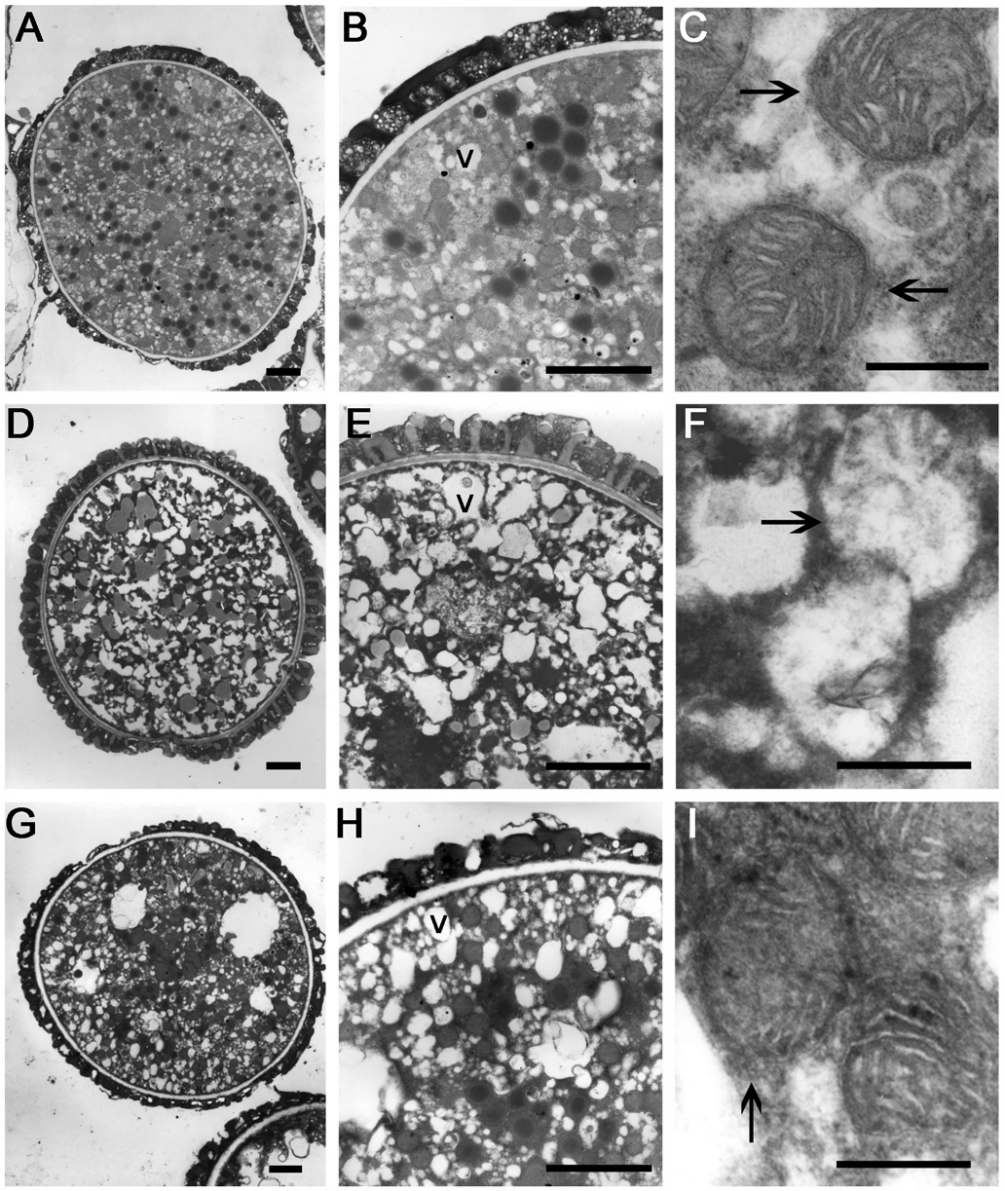
Vesicles and mitochondria in *kinβγ*/+ pollen. (A-C) Wild-type mature pollen grain. (B) and (C) The magnification of the pollen grain in (A) showing the vesicles (B) and mitochondria (C, arrows), respectively. (D–F) Mature pollen grain of *kinβγ-1/+* with enlarged vesicles. (E) and (F) The magnification of the pollen grain in (D) showing the enlarged vesicles (E) and abnormal mitochondria (F, arrow). (G–I) Mature pollen grain of *kinβγ-2/+ qrt1/-* with enlarged vesicles. (H) and (I) The magnification of the pollen grain in (G) showing the enlarged vesicles (H) and abnormal mitochondria (I, arrow), respectively. V, vesicle; Bars, 5 μm in (A, B, D, E, G, H) and 0.2 μm in (C, F, I).

**Fig 4:**
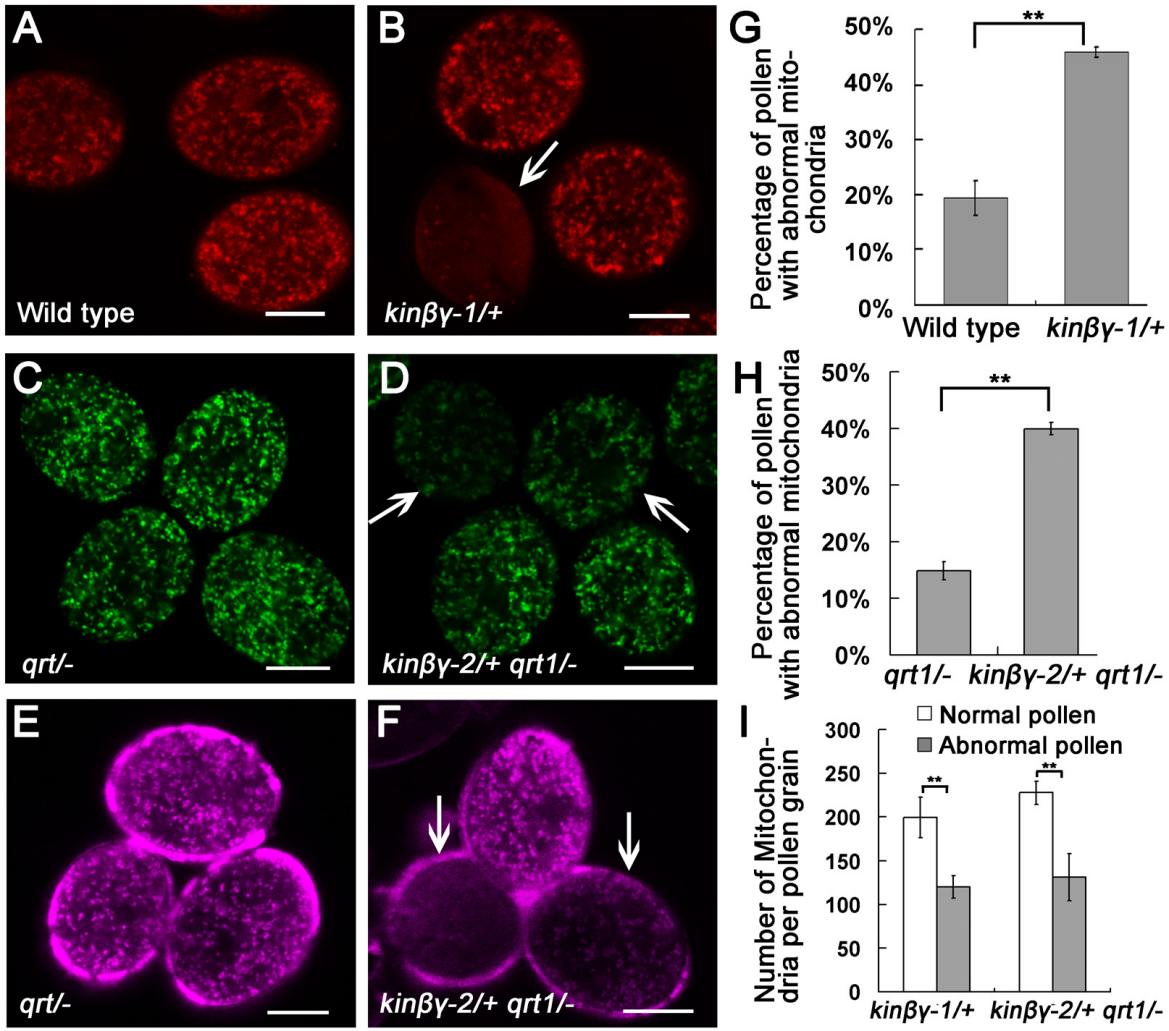
Fluorescence labeling analysis of pollen mitochondria in the *kinβγ/+* mutant. (A) and (B) VC-mtRFP labeling of mitochondria in the pollen of the wild type (A) and *kinβγ-1/+* (B), respectively. Arrow in (B) indicates a pollen grain with few mitochondria and the diffuse localization of VC-mtRFP in the cytoplasm. (C) and (D) VC-mtGFP labeling of the pollen mitochondria of *qrt1/-* (C) and *kinβγ-2/+ qrt1/-* (D), respectively. Arrows in (D) indicate pollen grains with few mitochondria and diffuse localization of VC-mtGFP in the cytoplasm. (E) and (F) MitoTracker Deep Red labeling of mitochondria in the pollen of *qrt1/-* (E) and *kinβγ-2/+ qrt1/-* (F), respectively. Arrows in (F) indicate mutant pollen grains with few mitochondria. (G) and (H) Percentages of pollen grains with abnormal mitochondria in the wild type, *kinβγ-1/+* (G), and *kinβγ-2/+* (H). Data were collected from three independent experiments. Asterisks indicate significant difference (Student’s t-test, P<0.01). (I) Average numbers of mitochondria in *kinβγ/+* pollen grains with normal and abnormal mitochondria. Only the mitochondria in the central optical sections of pollen grains observed under LSCM were counted for analysis. Data were collected from three independent experiments. Asterisks indicate significant difference (Student’s *t*-test, P<0.01). Bars, 10 μm.

To investigate the reason for the defective transmission of the *kinβγ* alleles through pollen, we observed the ultrastructure of pollen grains of the *kinβγ/+* mutants. Transmission electron microscopy (TEM) did not reveal any differences in pollen coat, exine, or intine morphology between wild-type and *kinβγ/+* mutant pollen grains (S3 Fig). However, many enlarged vesicles were found in the cytoplasm of some mutant pollen grains (approximately 47.4% in *kinβγ-1*/+ and 50.7% in *kinβγ-2*/+ *qrt1*/-); by contrast, many small vesicles were detected in the cytoplasm of mature wild-type pollen grains (Fig 3A, 3B, 3D, 3E, 3G and 3H). Interestingly, the mitochondria in wild-type pollen grains contained an electron-dense matrix and well-developed cristae inside the outer membrane (Fig 3C); however, mutant pollen grains with enlarged vesicles harbored mitochondria with incomplete outer membranes and disorganized cristae (Fig 3F and 3I). We then labeled the mitochondria in the pollen grains using two strategies: expressing mitochondria-targeted Vegetative Cell GFP/RFP (VC-mtGFP/RFP) [39] and staining the pollen grains with MitoTracker Deep Red. A number of *kinβγ-1/+* and *kinβγ-2/+ qrt1/-* pollen grains had reduced number of mitochondria (Fig 4A–4F). Approximately 19.4% and 46.0% of pollen grains harbored abnormal mitochondria in wild-type and *kinβγ-1/+* plants expressing VC-mtRFP compared with 14.9% and 40.0% in *qrt1/-* and *kinβγ-2/+ qrt1/-* plants expressing VC-mtGFP, respectively (Fig 4G and 4H). Moreover, diffuse VC-mtG(R)FP signals were detected in the cytosol of these abnormal pollen grains, or the mitochondria exhibited weaker fluorescence after labeling with MitoTracker Deep Red (Fig 4B, 4D and 4F). Furthermore, we determined the number of mitochondria in mutant pollen grains labeled with VC-mtG(R)FP by counting the mitochondria in a single central optical section via laser scanning confocal microscopy (LSCM). As shown in Fig 4I, there were approximately 120 and 131 mitochondria in the abnormal pollen grains of the *kinβγ-1/+* and *kinβγ-2/+ qrt1/-* mutants, respectively; by contrast, there were approximately 200 and 228 mitochondria in the normal pollen grains of the *kinβγ-1/+* and *kinβγ-2/+ qrt1/-* mutants, respectively.

Since KINβγ and the α-catalytic KIN10 and KIN11 subunits can form an active complex [37], we determined whether the defective mitochondrion phenotype was related to the KINα subunit by employing an amiRNA strategy to reduce *KIN10* and *KIN11* expression in pollen [40]. We generated a late anther tomato gene52 (Lat52)::amiRNA-*KIN10*,*11* construct and transferred it into *Arabidopsis*. Twenty-seven transgenic lines were obtained, four of which showed different expression levels of *KIN10*,*11* were chosen for further analysis, including examination of pollen viability, morphology, and mitochondria. Green pollen grains were identified in the transgenic lines after Alexander staining, indicating that the knockdown of *KIN10* and *KIN11* resulted in some pollen abortion (S4A Fig). Under SEM, some pollen grains of the transgenic lines had sunken surfaces, which were similar to those of the *kinβγ* mutant (S4B Fig). Furthermore, there were fewer mitochondria in some pollen grains of the transgenic lines, as revealed by MitoTracker Deep Red labeling (S4C Fig), which was also observed in the *kinβγ* mutant. The similar pollen phenotypes in both these lines suggest that the *KINβγ* works in coordination with *KIN10* and *KIN11* in SnRK1 complex for the regulation of *Arabidopsis* pollen development and mitochondrion biogenesis.

**Fig 5:**
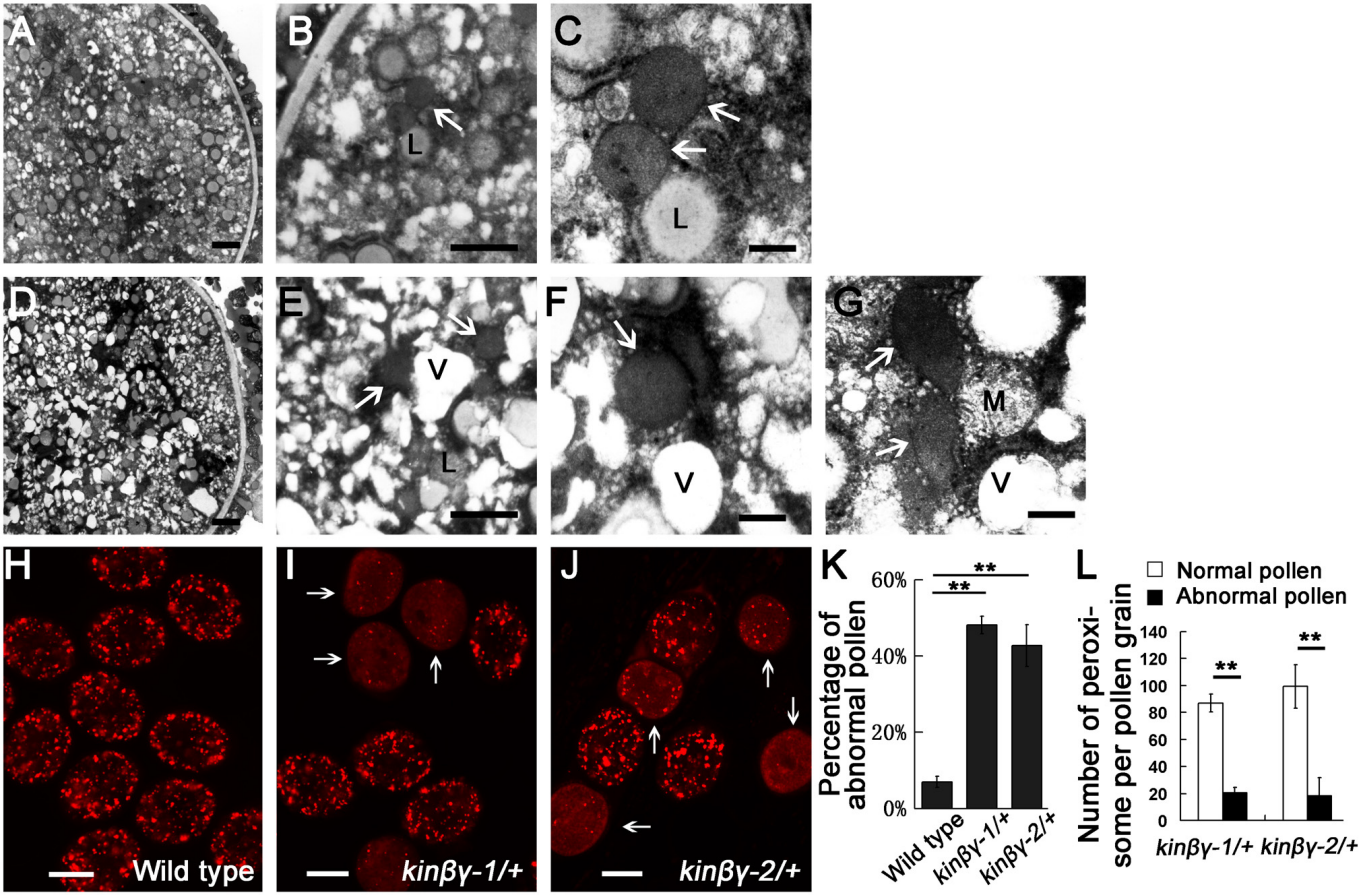
Peroxisomes in the pollen of *kinβγ/+* and the wild type. (A–C) Wild-type pollen under TEM. (B) and (C) The magnification of the pollen grain in (A), showing normal peroxisomes (arrows). (D–G) The *kinβγ-2* pollen under TEM. (E, F and G) The magnification of the pollen grain in (D), showing peroxisomes with difficult-to-detect membranes (arrows, in E and F) or irregular morphology (arrows, in G). (H–J) Pollen labeled with mCherry-PTS1 in the wild type (H), *kinβγ-1/+* (I), and *kinβγ-2/+* (J), showing the pollen grains with reduced numbers of peroxisomes (arrows) and diffused mCherry-PTS1 signal. (K and L) Statistical analysis of pollen grains with reduced numbers of peroxisomes (K) and the average number of peroxisomes in normal and abnormal pollen grains in *kinβγ/+* (L); only peroxisomes in the central optical sections of pollen grains were counted for analysis. Data were collected from three independent experiments. Asterisks indicate significant difference (Student’s *t*-test, P<0.01). L, lipid; M, mitochondrion; V, vesicle. Bars, 1 μm in (A, B, D, E), 200 μm in (C, F, G), and 10 μm in (H, I, J).

The mitochondria and peroxisomes are metabolically linked and share components of division machinery [41]. Thus, we examined the ultrastructure and the number of peroxisomes in *kinβγ/+* pollen grains using 3,3'-diaminobenzidine tetrahydrochloride and fluorescent protein labeling, respectively. In the mutant pollen grains, some peroxisomes lacked an obvious membrane or exhibited irregular morphology compared with the round peroxisomes with single-layered membranes observed in wild-type pollen grains (Fig 5A–5G). We then labeled the peroxisomes in the pollen grains using the peroxisome-targeted marker mCherry-PTS1 [16]. In the *kinβγ/+* mutant, many pollen grains harbored few peroxisomes and exhibited diffuse, red fluorescent mCherry-PTS1 signals in the cytoplasm (Fig 5H–5J). Approximately 48.0% of the pollen grains in *kinβγ-1/+* and 43.7% in *kinβγ-2/+* exhibited abnormal fluorescence patterns in their peroxisomes; by contrast, only 6.8% of wild-type pollen produced abnormal mCherry-PTS1 signals (Fig 5K). In the abnormal pollen grains of the *kinβγ-1/+* and *kinβγ-2/+* mutants, there were approximately 20 and 18 peroxisomes, respectively, in a single optical section under LSCM, whereas there were approximately 87 and 99 peroxisomes in the normal pollen grains of the *kinβγ-1/+* and *kinβγ-2/+* mutants, respectively (Fig 5L). These results indicate that the *kinβγ* mutation led to defects in the structure and biogenesis of mitochondria and peroxisomes in pollen grains.

### Pollen germination on stigma is defective in the *kinβγ/+* mutant

**Fig 6:**
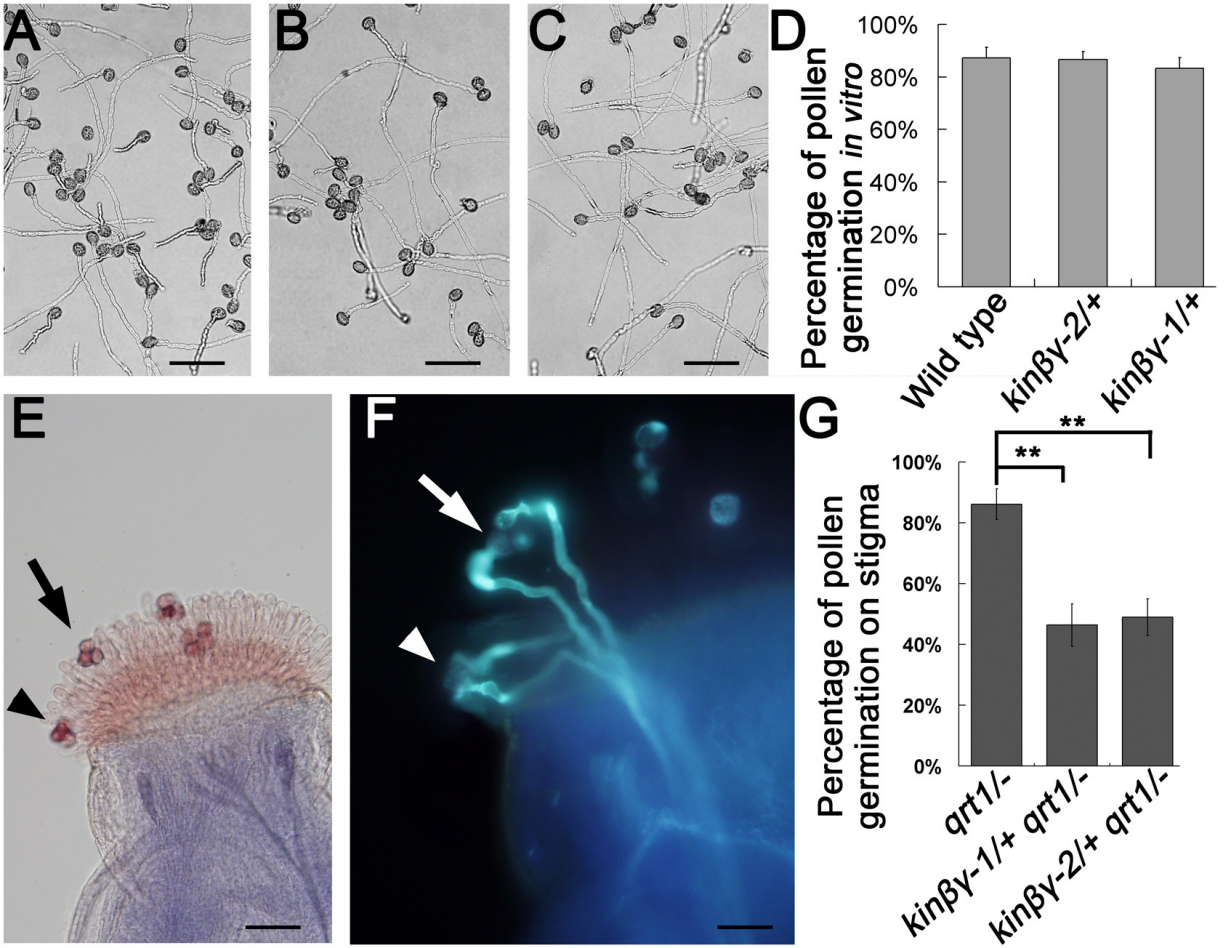
Pollen germination of the *kinβγ* mutant in vitro and in vivo. (A-C) Pollen germination of the wild type (A), *kinβγ-1/+* (B), and *kinβγ-2/+* (C) on medium. (D) Percentage of pollen germination of the wild type, *kinβγ-1/+* and *kinβγ-2/+*. (E) and (F) Pollen germination of *kinβγ-2/+ qrt1/-* on the stigma. (E) A bright field photograph. (F) A fluorescence photograph of amplified (E) labeled with aniline blue. Arrows and arrowheads indicate two *qrt1/-* tetrad pollen grains on the stigma. (G) Statistical analysis of the pollen germination of *qrt1/-*, *kinβγ-1/+ qrt1/-*, and *kinβγ-2/+ qrt1/-* on wild-type stigmas. Data were collected from three independent experiments. Asterisks indicate significant difference (Student’s *t-*test, P<0.01). Bars, 60 μm in (A–C, E); 40 μm in (F).

To further examine the pollen defects in the *kinβγ*/+ mutant, we performed both *in vitro* and *in vivo* pollen germination analyses. The pollen germination and pollen tube morphology of the *kinβγ*/+ mutant were no different from those of the wild type when cultured on germination medium (Fig 6A–6D). We also hand-pollinated the pollen from *qrt1*/-, *kinβγ-1*/+ *qrt1*/-, and *kinβγ-2*/+ *qrt1*/- onto emasculated wild-type stigmas, respectively, and analyzed pollen germination after 4 h using aniline blue staining. The pollen germination ratios of the *kinβγ-1*/+ *qrt1*/- and *kinβγ-2*/+ *qrt1*/- mutants were 46.4% and 49.0%, respectively, but the ratio of *qrt1*/- pollen was approximately 86.1% (Fig 6E–6G). Therefore, the presence of the *kinβγ* mutation caused more pollen to exhibit defective germination on the stigma. These results indicate that the *in vivo* germination of pollen grains with the *kinβγ* mutation was compromised, although the *in vitro* germination of mutant pollen did not show significant abnormality compared with that of wild-type pollen.

### The adhesion and hydration of *kinβγ* mutant pollen on the stigma is compromised

**Fig 7:**
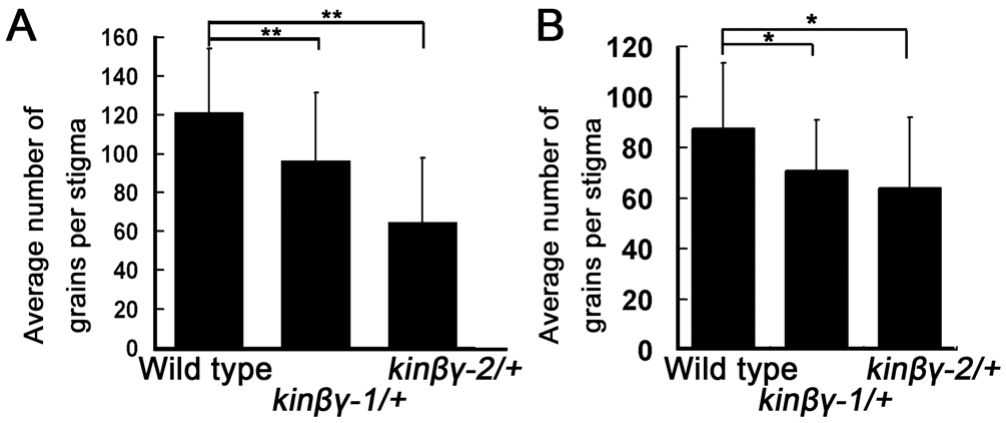
Statistical analysis of *kinβγ/+* pollen adhesion on the stigma. The number of pollen grains of the wild-type, *kinβγ-1/+*, and *kinβγ-2/+* on washed stigmas were counted in the natural pollination (A) and the hand pollination (B). Data were collected from three independent experiments. Nine or ten stigmas were analyzed in each experiment. Double asterisks and asterisk indicate significant difference at P<0.01 and P<0.05 (Student’s *t*-test), respectively.

Since pollen adhesion and hydration are required for germination on the stigma, we examined whether adhesion and hydration of pollen on the stigma were affected in the *kinβγ* mutant. To analyze pollen adhesion, we collected *kinβγ-1/+*, *kinβγ-2/+*, and wild-type pistils at 9 h after flowering and counted the number of pollen grains on stigmas that had been washed with 0.01% NP-40 solution, which does not disturb the binding of *Arabidopsis* pollen on the stigma but removes weakly adhering pollen [7]. The average number of pollen grains on the mutant stigmas (97 in *kinβγ-1/+*, 65 in *kinβγ-2/+*) was much less than that on wild-type stigmas (121 in the wild type) after washing (Fig 7A). In addition, we hand-pollinated the pollen grains of the wild-type, *kinβγ-1/+*, and *kinβγ-2/+* onto the wild-type stigmas, and collected the pollinated stigmas after 4 h, respectively. Then, the pollen grains on the stigmas washed with 0.01% NP-40 solution were counted. The average number of the pollen grains of the mutants (71 in *kinβγ-1/+*, 62 in *kinβγ-2/+*) was also less than that of the wild type (88 in the wild type) (Fig 7B). The results indicate that the *kinβγ* mutations result in defective pollen adhesion on the stigmas.

**Fig 8:**
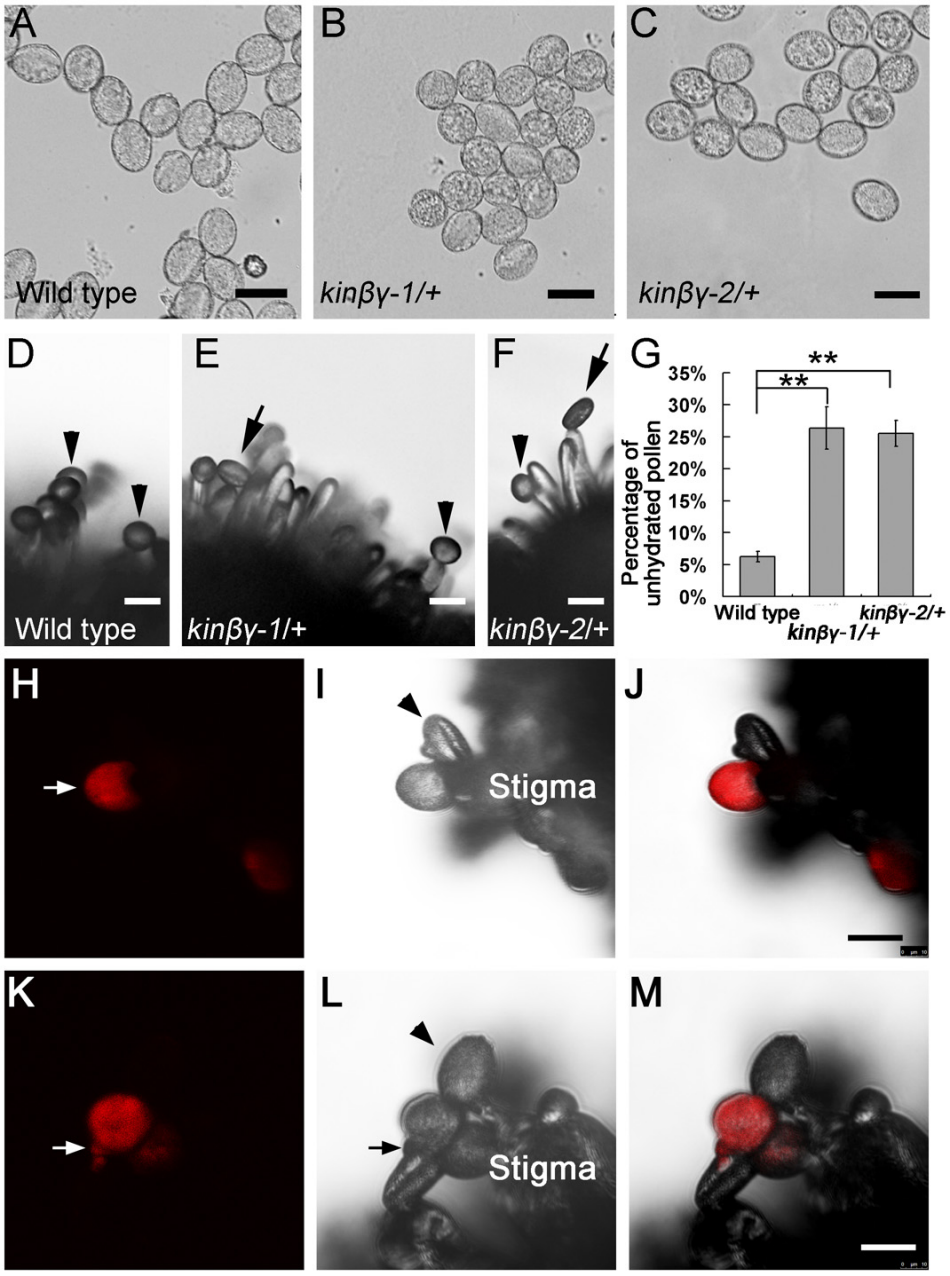
Pollen hydration in vitro and in vivo. (A–C) Pollen of the wild type (A), *kinβγ-1/+* (B) and *kinβγ-2/+* (C) at 5 min after scattering onto the medium. (D-F) Pollen grains of the wild type (D), *kinβγ-1/+* (E), and *kinβγ-2/+* (F) at 5 min after pollinated on the wild-type stigmas. Arrowheads and arrows indicate hydrated and unhydrated pollen grains, respectively. (G) Statistical analysis of the number of unhydrated pollen grains of the wild type and *kinβγ/+* at 5 min after pollinated on the wild-type stigmas. Data were collected from three independent experiments. Asterisks indicate significant difference (Student’s *t*-test, P<0.01). (H–J) Hydration of the *kinβγ-2/+ dsred/+ qrt1/-* pollen grains on the wild-type stigmas at 5 min after hand-pollination. Arrow in (H) and arrowhead in (I) indicate a hydrated (red fluorescence signal) and an unhydrated (no red fluorescence signal) pollen grains in a tetrad, respectively. (H–J) The photographs of fluorescence channel, bright-field channel, and the merged channel, respectively. (K–M) Germination of the *kinβγ-2/+ dsred/+ qrt1/-* pollen grains on the wild-type stigmas at 25 min after hand-pollination. Arrows in (K, L) and arrowhead in (L) indicate a pollen tube with red fluorescence signal and a hydrating pollen grain without red signal, respectively. (K), (L), and (M) The photograph of fluorescence channel, bright-field photograph, and the merged photograph, respectively. No mounting medium was used in the preparation of the temporary slides for the observations and photographs of (D–F, H–M). Bars, 20 μm.

To determine whether pollen hydration was disturbed in the *kinβγ* mutant, we evaluated the *in vitro* and *in vivo* hydration of wild-type and mutant pollen grains. First, we observed pollen hydration on germination medium and found that the mutant and wild-type pollen became rounded at 5 min after hydration. No difference in morphology was identified in both mutant and wild-type pollen grains (Fig 8A–8C). We then investigated pollen hydration on the stigma by observing mutant and wild-type pollen grains on wild-type stigmas. Because the mutant pollen grains could hydrate in the water-contained environment, no water was added in the mounting medium on slide for in vivo hydration and germination analysis. The wild-type pollen completed the hydration process within 5 min on the stigma; however, only some pollen grains of the *kinβγ-1/+* and *kinβγ-2/+* mutants became hydrated (Fig 8D–8F). Statistical analysis revealed that approximately 25% of the pollen grains of the *kinβγ-1/+* and *kinβγ-2/+* mutants failed to become hydrated on the stigma; by contrast, <7% of wild-type pollen remained unhydrated (Fig 8G). The fluorescent-tagged line 1262 (FTL1262) is an *Arabidopsis* transgenic line expressing LAT52::*DsRed* under *qrt1* background in which *DsRed* is a single-locus insertion in chromosome 1 near the *KINβγ* locus (At1g09020) [42]. We obtained *kinβγ-2/+ dsred/+ qrt1/-* plants by crossing *kinβγ-2/+ qrt1/-* and *DsRed/+ qrt1/-* (FTL1262) plants, in which the red marker co-segregates with the wild-type allele of *KINβγ*. We hand-pollinated the *kinβγ-2/+ dsred/+ qrt1/-* grains on the wild-type stigmas and observed their hydration and germination at 5 min and 25 min after pollination under LSCM, respectively. As shown in Fig 8H–8M, the pollen grains with red fluorescence signal could hydrate and germinate on the wild-type stigmas. Occasionally, the pollen grain without red signal could hydrate on the stigma (Fig 8M).

**Fig 9:**
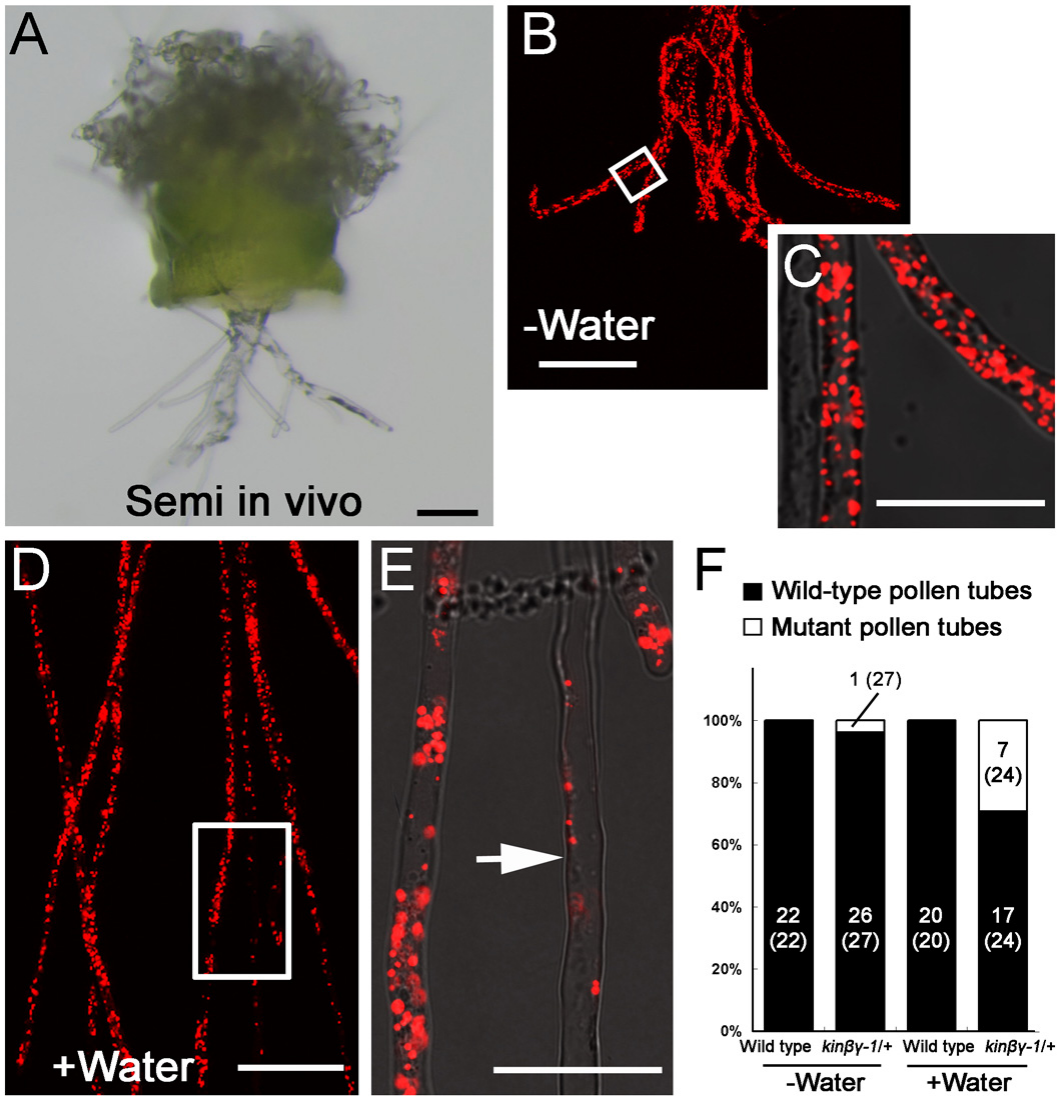
Germination of *kinβγ-1* pollen on a wild-type stigma moistened with water in semi-*in vivo* system. (A) A semi-*in vivo* pollen tube growth system. (B) Pollen tubes of *kinβγ-1/+* expressing VC-mtRFP penetrated the pistil tissue without added water. (C) Magnification of the box in (B). (D) Pollen tubes of *kinβγ-1/+* expressing VC-mtRFP penetrated the pistil tissue after adding water onto the stigma. (E) Magnification of the box in (D), showing a mutant *kinβγ-1* pollen tube (arrow). (F) Counting the pollen tubes of the wild type and the *kinβγ-1/+* that penetrated pistil tissue with and without added water. The number in the columns represents the number of pollen tubes. The number in the brackets is the total number of counted pollen tubes. Bars, 50 μm in (A), (B) and (D), and 25 μm in (C) and (E).

Hydration is a prerequisite for pollen germination on the stigma. To determine whether compromised hydration caused the defective germination of *kinβγ* pollen on stigma, we analyzed the role of water in the germination of mutant pollen on stigmas. We pollinated *kinβγ-1/+* mutant pollen expressing *VC-mtRFP* onto wild-type stigmas in the presence of water in a semi-*in vivo* system [43] and identified mutant pollen tubes that had grown through pistil tissues based on their reduced number of mitochondria (Fig 9A–9E). As shown in Fig 9F, 7 of 24 pollen tubes from *kinβγ-1/+ VC-mtRFP* that penetrated the pistil tissue in the added water condition exhibited the reduced number of mitochondria; by contrast, only 1 of 27 pollen tubes that had penetrated the no water-added stigmas was a mutant pollen tube. No pollen tube showed the reduced number of mitochondria when the wild-type pollen grains were pollinated on the stigma with and without added water (Fig 9F). These results suggest that defective hydration, at least in part, results in the compromised germination of *kinβγ* pollen on stigmas.

### Reactive oxygen species levels are reduced in *kinβγ* pollen

**Fig 10:**
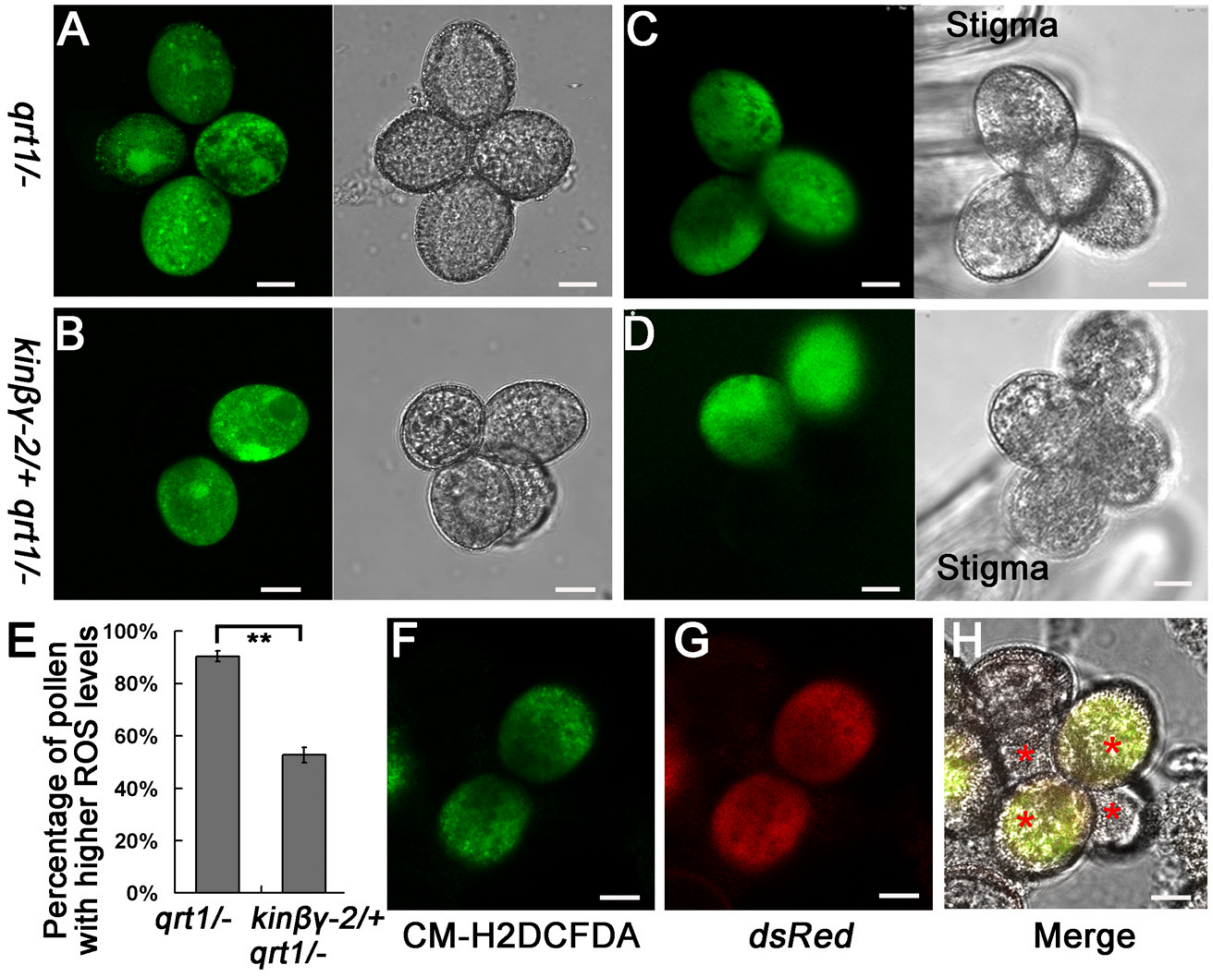
ROS analysis in pollen grains of *qrt1/-* and *kinβγ-2/+ qrt1/-*. (A–D) CM-H2DCFDA labeling of ROS in mature pollen grains (A, B) and pollinated grains (C, D) of *qrt1/-* (A, C) and *kinβγ-2/+ qrt1/-* (B, D). (E) Statistical analysis of pollen with high ROS levels in *qrt1/-* and *kinβγ-2/+ qrt1/-*. ImageJ software was used for the analysis of the images. First, the format of images was changed into 8-bit and inverted into image color. Then, the threshold levels of the images were set between 0.21 and 0.02. The pollen grains were marked by red color with high ROS levels. Data were collected from three independent experiments. Asterisks indicate significant difference (Student’s *t*-test, P<0.01). (F–H) ROS labeled by CM-H2DCFDA in *kinβγ-2/+ dsred/+ qrt1/-* pollen grains. Green fluorescence signals (F) and red fluorescence signals (G) originate from the CM-H2DCFDA and *LAT52*::*DsRed*, respectively. (H) Merged image of (F) and (G). Asterisks in (H) indicate *qrt1/-* tetrad pollen. Bars, 10 μm.

The mitochondria, peroxisomes, and chloroplasts are the major sites of intracellular ROS production [44]. We hypothesized that ROS levels might be reduced in mutant *kinβγ* pollen because of its defects in mitochondrial and peroxisomal biogenesis. Thus, we investigated the ROS levels in pollen *in vitro* by labeling it with chloromethyl derivative of 2′,7′-dichlorodihydrofluorescein diacetate (CM-H2DCFDA), which revealed high ROS levels in most *qrt1/-* pollen grains (90.3%); however, only approximately half of the *kinβγ-2/+ qrt1/-* pollen grains (52.5%) showed high ROS levels (Fig 10A, 10B and 10E). When the pollen grains were pollinated onto the stigma (*in vivo*), a similar phenotype, i.e., high ROS levels in pollen, was observed (Fig 10C and 10D). We also detected the ROS levels by CM-H2DCFDA-labeling in the pollen from *kinβγ-2/+ dsred/+ qrt1/-* plants: green fluorescent signals in these plants indicated that the pollen had high ROS levels, and red signals represented the wild-type pollen. Both red and green fluorescent signals were detected together in two pollen grains of a pollen tetrad (Fig 10F–10H), confirming that the ROS levels in pollen grains with the *kinβγ* mutation were significantly reduced.

**Fig 11:**
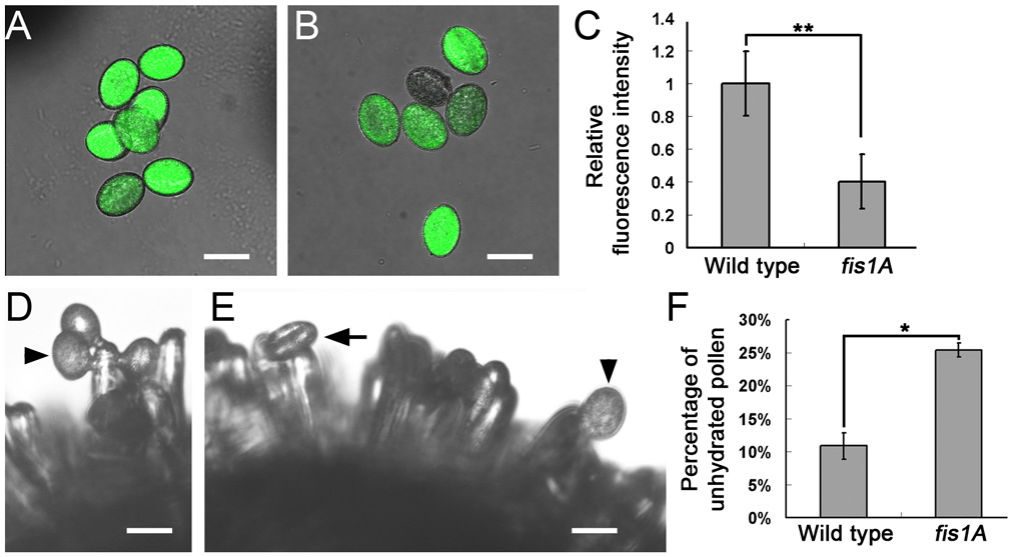
ROS levels and in vivo hydration of the pollen grains of the *fis1A* mutant. (A, B) CM-H2DCFDA labeling to determine the ROS levels of pollen grains in the wild type (A) and *fis1A* mutant (B). (C) Relative ROS levels in pollen grains of the wild type and *fis1A* mutant. Data were collected from three independent experiments. Double asterisks indicate significant difference at P<0.01 (Student’s *t*-test). (D, E) In vivo hydration of the pollen grains of the wild type (D) and *fis1A* mutant (E). (F) Percentages of the unhydrated pollen grains of the wild type and *fis1A* mutant. Data were collected from three independent experiments. Asterisk indicates significant difference at P<0.05 (Student’s *t*-test). Arrowheads and arrow indicate the hydrated and unhydrated pollen grains in (D) and (E). Bars, 20 μm in (A, B, D, and E).

*Arabidopsis* FISSION1A (FIS1A) is a mitochondrion- and peroxisome-targeted protein required for the division of both organelles. In the knockout mutant of *FIS1A* (*fis1A*), the numbers of mitochondria and peroxisomes are less than those of the wild type [45]. To determine whether the defect in mitochondrial and peroxisomal biogenesis is correlated with the reduced ROS level, we detected the ROS levels and hydration of the pollen grains from the *fis1A* mutant plants. The ROS levels in the pollen grains of *fis1A* were significantly decreased compared with those of the wild type (Fig 11A–11C). Furthermore, about 25.4% of the *fis1A* pollen grains on the stigmas could not complete their hydration for 5 min; in contrast, only 10.5% wild-type pollen grains did not hydrate for 5 min (Fig 11D–11F). These results confirmed that the defect in the mitochondrial and peroxisomal biogenesis contributes to the reduced levels of ROS in pollen grains and the compromised hydration of pollen grains.

### ROS is essential for pollen hydration on stigmas

**Fig 12:**
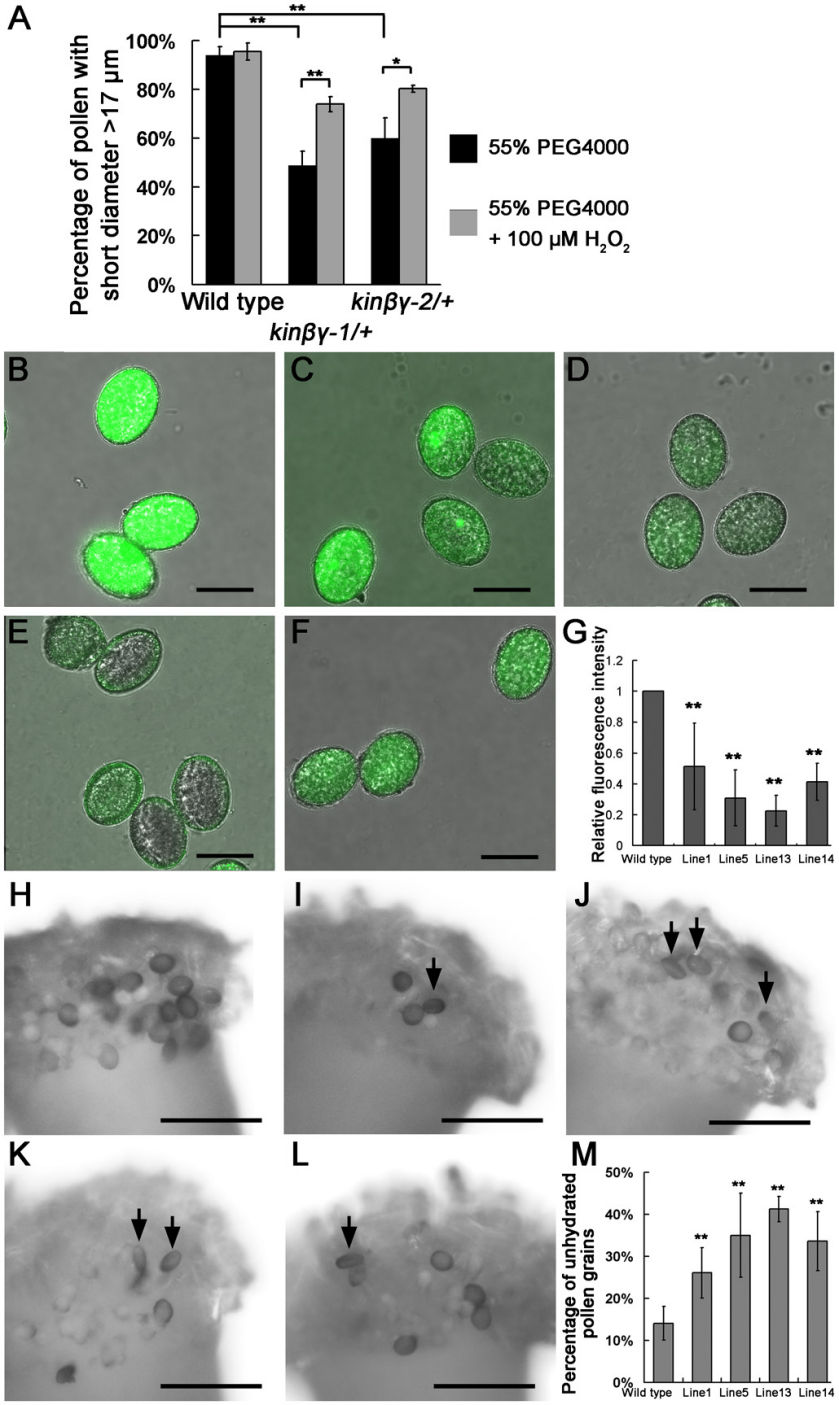
ROS functions in pollen hydration. (A) H_2_O_2_ promotes water absorption of *kinβγ/+* pollen *in vitro*. 55% PEG4000 was added to the pollen medium (please see “Methods and Materials” in detail) to mimic a low water-potential environment. At 15 min after the pollen grains were scattered in the medium with or without 100 μM H_2_O_2_, the short diameters of pollen grains were measured. Data were collected from three independent experiments. Double asterisks and single asterisk indicate significant difference at P<0.01 and <0.05 (Student’s *t*-test), respectively. (B–F) CM-H2DCFDA labeling to determine the ROS levels of pollen from the wild type (B), and the transgenic lines 1 (C), 5 (D), 13 (E), and 14 (F) overexpressing *Arabidopsis CAT3*. (G) Relative ROS levels of pollen grains labeled with CM-H2DCFDA in different transgenic lines overexpressing *Arabidopsis CAT3*. ** indicates significant difference. (H–L) Hydration of pollen grains of the wild type (H), and the transgenic lines 1 (I), 5 (J), 13 (K), and 14 (L) overexpressing *Arabidopsis CAT3* on the wild-type stigmas at 10 min after pollination. (M) Percentage of unhydrated pollen grains on the surface of wild-type stigmas at 10 min after pollination. ** indicates the significant difference. Bars, 20 μm in (B–F), and 100 μm in (H–L).

To determine whether the reduced ROS levels resulted in defective pollen hydration, we determined the water absorption capacity of mutant pollen in the modified pollen germination medium containing H_2_O_2_ (please see “Methods and Materials” in detail). We added polyethylene glycol 4000 (PEG4000) in medium to mimic a low water potential environment and investigated pollen hydration by measuring the short diameters of pollen grains at 15 min. The difference of pollen percentages with short diameter <17 μm between the wild type and the *kinβγ-2/+* mutant in the 55% PEG4000 is more obvious than that in 35% PEG4000 (S6 Fig). Furthermore, we found that 100 μM H_2_O_2_ was the most appropriate concentration for the analysis of the pollen hydration of *kinβγ-1* mutant in the 55% PEG4000-containing medium (S6 Fig). Thus, we used the medium with 55% PEG4000 and 100 μM H_2_O_2_ for further analysis of pollen hydration. The short diameter of approximately 94.0% of the wild-type pollen grains was >17 μm; by contrast, only 48.8% and 59.9% of the pollen grains from *kinβγ-1/+* and *kinβγ-2/+*, respectively, had a short diameter >17 μm. However, when we added 100 μM H_2_O_2_ in the medium, the percentage of pollen grains with a short diameter >17 μm was 95.43%, 73.97%, and 80.30% in wild-type, *kinβγ-1/+*, and *kinβγ-2/+* pollen, respectively (Fig 12A), suggesting that ROS promote water absorption in *kinβγ* mutant pollen.

To determine whether ROS are involved in the regulation of pollen hydration on the stigma, we reduced the ROS levels in the pollen grains by overexpressing *Arabidopsis* CATALASE 3 (CAT3, At1g20620), which catalyzes the decomposition of hydrogen peroxide to water and oxygen [46]. We produced a *LAT52*::*CAT3* construct and obtained 22 *Arabidopsis* transgenic lines. There were some shrunken and nonviable pollen grains in four lines, suggesting the overexpression of *CAT3* in these lines resulted in abnormal pollen development (S5 Fig). Then, four transgenic lines that showed different expression levels of *CAT3* and did not exhibit abnormal pollen morphology were chosen for the following analysis (S5 Fig). Alexander staining revealed that the pollen viability of these transgenic lines was similar to that of the wild-type control (S5 Fig). The ROS levels of the pollen grains in these four lines were significantly reduced, as revealed by CM-H2DCFDA labeling and semiquantitative analysis (Fig 12B–12G). As expected, a number of pollen grains in the transgenic lines did not become rounded at 10 min after pollination; by contrast, the majority of wild-type grains (>85%) on the stigmas were round at this time (Fig 12H–12M). Thus, ROS levels in pollen are involved in the hydration of pollen on stigmas.

## Discussion

### KINβγ regulates the biogenesis of mitochondria and peroxisomes

The KINβγ subunit is a plant-specific component of the SnRK1 heterotrimeric complexes. This subunit contains four CBS domains, as well as a GBD/KIS domain that is normally identified in canonical β subunits [32]. In *Arabidopsis*, KINβγ can form an active complex with either of the catalytic subunits (KIN10, KIN11) [37]. It was recently demonstrated that the carbohydrate-binding module in the GBD/KIS domain of *Arabidopsis* KINβγ does not bind to starch or similar oligo/polysaccharides [23], although the GBD/KIS domains in the γ subunits of the SNF1/AMPK complex in yeast and mammals can bind to glycogen to regulate complex processes. Two *Arabidopsis* leucine-rich proteins that interact with KINβγ are involved in plant–pathogen interactions [34]. Here, we found that the *kinβγ* mutation led to defective biogenesis of mitochondria and peroxisomes in *Arabidopsis* pollen, which resulted in compromised pollen germination on the surface of the stigma. Indeed, a previous study showed that the homozygous knockout mutant of KINβγ in *Arabidopsis* could not survive [32], perhaps due to the abnormal biogenesis of its mitochondria and peroxisomes. In this study, we found the knockdown expression of the *Arabidopsis KIN10*,*11* also led to abnormal pollen morphology and reduced mitochondrial number in pollen, similar to the phenotypes of *kinβγ* knockout mutant. Additionally, the pollen viability was severely impaired in the knock-down mutant of the *KIN10*,*11*, which is consistent with the phenotypes of the small, non-functional pollen of the barley (*Hordeum vulgare*) transgenic lines expressing an antisense construct of barley *SnRK1b* (*BKIN12*), encoding a homolog of SnRK1 kinase subunit [24]; in contrast, *kinβγ* mutations did not result in obvious loss of pollen vitality in Alexander staining. Thus, it is likely that other γ subunits of SnRK1 complex also play roles for the activity regulation of the kinase subunit in determining pollen viability.

In mammals, AMPK promotes the biogenesis of mitochondria through the action of the transcriptional coactivator PGC-1α (peroxisome proliferator-activated receptor gamma coactivator 1-alpha) [47] and destroys defective mitochondria through unc-51-like autophagy activating kinase 1 (ULK1)-dependent autophagy [48, 49], by which new, functional mitochondria replace the defective mitochondria [19]. However, the number of mitochondria in mouse liver cells significantly increases under AMPK deficit [48], which differs from our observation that defective expression of *KIN10/11* and *KINβγ* reduced the number of mitochondria. These results suggest that the plant SnRK1 complex utilizes different regulatory mechanisms for mitochondrial proliferation from that of its counterparts in mammals, which is consistent with the finding that *Arabidopsis* SnRK1 is an atypical member of the SNF1/AMPK/SnRK1 family [23]. Identifying SnRK1 complex-interacting molecules in plants might help elucidate the different ways in which the SnRK1 complex regulates the biogenesis of mitochondria and peroxisomes. There is some evidence that KIN10/11 interacts with transcription factors and other proteins in *Arabidopsis* [50–52]. Recently, the proteins interacting with *Arabidopsis* KIN10/11 were identified using a yeast two-hybrid system [53], in which several proteins containing a domain of unknown function (DUF) 581, such as DUF581-5, DUF581-13, DUF581-18, and DUF581-19, are mitochondrial-localized proteins based on the analysis at subcellular localization database for *Arabidopsis* proteins (http://suba.plantenergy.uwa.edu.au/) [54]. Whether these mitochondrial-localized proteins mediate the SnRK1 complex regulation in the biogenesis of mitochondria and peroxisomes in *Arabidopsis* remains to be investigated.

### ROS signaling in pollen mediates pollen–stigma interactions

After pollen grains land on the stigma during pollination, the ability of the pollen grains to germinate in dry stigma species depends on their interactions with the papilla cells, a highly regulated process [2]. The pollen exine and coat are the first components that come in contact with the stigmatic papilla, which is involved in regulating pollen adhesion, hydration, and germination on the surface of the stigma. Many molecules located in the pollen coat function in these interaction processes, such as long-chain lipids [9], GRP17 [13], and EXL4 [14] in *Arabidopsis*, xylanase in maize [15], and S-locus protein 11 (SP11) in *Brassica* [55]. In general, the components of the exine and coat originate in the tapetum, the innermost sporophytic cell layer of the anther [56]. In this study, we did not detect any structural changes in the exine or coat of *kinβγ* mutant pollen grains, although the adhesion and hydration of the mutant pollen on the surface of the stigma were compromised. Nonionic detergent-washing analysis previously demonstrated that the lipophilic molecules in the exine mediate pollen adhesion on the stigma [7]. We found that NP-40 disrupted the adhesion of *kinβγ* mutant pollen grains to the stigma, suggesting that some lipophilic molecules localized on the surface of *kinβγ* mutant pollen were altered, or perhaps their functions varied compared with the wild type. However, the wild-type pollen produced by the *kinβγ/+* mutant did not exhibit detectable changes in hydration or germination compared with that from wild-type plants, suggesting that some molecules in the exine and coat involved in pollen adhesion and hydration are regulated by factors in the pollen rather than in sporophytic tissue. The internal molecules in the exine and pollen coat and molecules involved in pollen signaling may not readily interact during the interactions between the pollen and stigma because they do not directly come in contact with each other. In the present study, we found that ROS signaling originating from the interior of pollen grains is important for the interactions between the pollen and stigma. A study of the *Arabidopsis dayu* mutant demonstrated that jasmonic acid signaling in the pollen grain controls pollen germination on the stigma [16]. *DAYU* and *KINβγ* are expressed in the vegetative pollen cell. These findings indicate that the internal signaling of pollen grains is involved in pollen–stigma interactions.

ROS, which are ubiquitous regulatory molecules, have been implicated in many pollen-related processes in sexual plant reproduction, such as tapetum and pollen development [57–59], *in vitro* pollen germination [60], pollen tube tip growth [61–63], pollen tube rupture for sperm release [64], and the self-incompatibility response [65]. Nicotinamide adenine dinucleotide phosphate oxidase, which is localized to the plasma membrane, is involved in ROS production, except in rice tapetum, where the mitochondria are essential for maintaining ROS homeostasis [58]. Our results show that the mitochondria and peroxisomes contribute to ROS production in mature pollen of the *kinβγ* and *fis1A* mutants, which regulates the interactions between the pollen and stigma during pollination. However, the regulatory mechanisms remain to be investigated. A possible role for internal signaling in pollen, which functions in pollen–stigma interactions, is the control of the addition of the pollen coat and exine molecules; indeed, some maize pollen coat proteins are thought to be synthesized within the pollen interior [66]. We propose that ROS signaling might control the addition of lipophilic or other molecules in the exine or on the surface of pollen, which is important for pollen–stigma interactions. Alternatively, internal pollen signaling might give rise to the transport of some mobile signaling molecules from the pollen interior to the surface of pollen grains to regulate pollen–stigma interactions following pollination. Therefore, identifying potential ROS targets that regulate the interactions between the pollen and stigma is important for elucidating the role of ROS signaling in pollination interactions. ROS signaling is intimately linked to the regulation of Ca^2+^ and K^+^ levels and, consequently, turgor homeostasis in pollen tubes [62–64]. The Ca^2+^ and K^+^ levels in pollen might be regulated by internal pollen ROS signaling, which adjusts the water potential and turgor in pollen during its hydration on the stigma. Additionally, we found some pollen grains of the *kinβγ* mutants showing the shrunken morphology under SEM. However, this abnormal morphology could not be detected after pollen hydration. In deed, various degrees of defective ultrastructures of vesicles and mitochondria in the mutant pollen were observed in our study. The severe defect of the ultrastructures of mutant pollen grains might correlate with their severe defect in osmotic adjustment ability. Thus, an artificial dry environment was imposed during the SEM sample preparation, which resulted in some of the mutant pollen showing the sunken surfaces under this condition.

Another way in which internal signaling in pollen may regulate pollen–stigma interactions is that it may initiate a signaling cascade to activate stigma cells during their interaction. For example, in *Brassica*, during self-incompatibility, SP11 is secreted from the pollen coat and interacts with its cognate *S*-receptor kinase in the papilla cell of the stigma to elicit the self-incompatibility response [55]. The pollen coat of *Brassicaceae* can induce the expression of Ca^2+^-ATPase 13, a Ca^2+^ transporter in the papillae cell that functions in the export of Ca^2+^ to compatible pollen tubes [67]. Similarly, the *Arabidopsis* pollen coat can induce the expression of many stigma genes, suggesting that the upregulation of these genes is most likely involved in pollen–stigma interactions. The stigma contains water on its surface, providing water for pollen hydration; the water is covered by a cuticle. However, it is currently unclear whether the stigma cuticle is constitutively permeable to water or if its permeability is modulated by pollen signaling [2]. Compatible pollen can induce the hydration and germination of normally incompatible pollen in mixed-pollen populations [9], suggesting that the permeability of the cuticle to water is regulated by pollen signaling. Thus, ROS signaling is essential for pollen hydration on the surface of the stigma, which might modulate the water permeability of the cuticle.

## Materials and Methods

### Plant materials and growth conditions

The *Arabidopsis thaliana* T-DNA insertion mutants GABI_346E09, SALK_074210 and *fis1A* were obtained from European Arabidopsis Stock Centre and Arabidopsis Biological Resource Center, respectively. Wild-type *Arabidopsis* (Columbia-0) and T-DNA insertion mutants were grown in a 22°C greenhouse under a 10-h light/14-h dark cycle (short-day condition) for 2 weeks and then a 16-h light/8-h dark cycle (long-day condition). The genotypes of the T-DNA insertion line plants were identified using a PCR-based method with the following primers: o8409, *kinβγ*-1LP, and *kinβγ*-1RP for GABI_346E09; and LBb1.3, *kinβγ*-2LP, and *kinβγ*-2RP for SALK_074210. All of the sequences of the primers are listed in the S3 Table.

### Pollen viability and germination analysis

The mature pollen grains or mature anthers were soaked in Alexander stain for 1–2 days to determine pollen viability. DAPI (1 μg/ml; w/v) (Sigma-Aldrich) was used to stain the nuclei of the pollen grains. Alexander stain, DAPI and aniline blue staining of pollen grain/tube were performed as previously described [68]. The semi *in vivo* and *in vitro* pollen germination assay was performed as described as Li et al. [16] and Xu et al. [69], respectively. All samples were observed and photographed under a BX51 microscope (Olympus, Tokyo, Japan).

### Pollen adhesion assay

The pollen adhesion assay was performed as described by Zinkl et al. [7] with minor modification. The self-pollinated pistils at the flowering day were collected at 7:00 PM. The hand-pollinated wild-type pistils which were emasculated two days before anthesis were collected at 4 hours after pollination. After washing with 0.05% NP-40 in 50 mM phosphate buffer (pH 7.4), the pistils were centrifuged for 30 s at 300 *g*. The adhering pollen grains on the washed pistils were photographed and counted under a BX51 microscope (Olympus, Tokyo, Japan).

### Pollen hydration assay

For the *in vivo* assay of pollen hydration, the stigmas of the emasculated flowers were hand pollinated with mature pollen grains in a greenhouse and observed after 5–10 min. For the *in vitro* assay of pollen hydration, the mature pollen grains were spread in the modified pollen germination medium (1 mM CaCl_2_, 1 mM Ca(NO_3_)_2_, 1 mM MgSO_4_, 0.01% (w/v) H_3_BO_3_, pH 7.0) by removing the sucrose and agar [69], at 28°C and observed after 15 min. To test the *in vitro* pollen hydration capacity, we used 35% and 55% (w/v) PEG4000 solution to mimic the low water potential environment. Fifteen min after the pollen grains incubated in the medium, the short-axis diameters of the pollen grains were measured. To analyze the role of ROS in pollen hydration of pollen *in vitro*, 0, 50, 100, and 150 μM H_2_O_2_ were added to the PEG4000-containing medium, and the short-axis diameters of the pollen grains were measured after 15 min. All pollen grains were observed under a BX51 microscope (Olympus, Tokyo, Japan) or LEICA TCS SP5 II laser scanning confocal microscope (Leica, Wetzlar, Germany).

### Fluorescent labeling of mitochondria and peroxisomes

Two approaches were used to label mitochondria. One was fluorescence staining using 500 mM MitoTracker Deep Red FM (Cat# M22426; Molecular Probes), which was excited with 633 nm laser and the signal were collected 650–700 nm. Another method for detecting mitochondria was using the *Arabidopsis* mitochondria marker lines—VC-mtGFP and VC-mtRFP—which were excited at 488 and 561 nm, and emissions were collected at 505–530 and 570–660 nm, respectively. The Lat52::mCherry-PTS1 transgenic line was used to label peroxisome. mCherry-PTS1 was excited by a 561 nm laser, and the emissions were observed at 600–630 nm. All images were captured using LEICA TCS SP5 II LSCM (Leica, Wetzlar, Germany). The numbers of mitochondria and peroxisomes in the central optical sections of the pollen grains were counted for the statistical analysis.

### Scanning and transmission electron microscopy (SEM and TEM)

Sample preparation and observation for routine SEM and TEM were performed as previously described [69]. To visualize the peroxisomes of the pollen under TEM, 3,3'-diaminobenzidine tetrahydrochloride (DAB) staining was used to detect the catalase activity, which marks the peroxisome, as described by Li et al. [16]. The anthers were fixed at 4°C for 10 h with 2.5% glutaraldehyde and 1% osmium tetroxide at 4°C for 5 h in 0.1 M cacodylate buffer (pH 7.2). After washing 3 times with cacodylate buffer, the anthers were incubated for 2 h at 28°C in the dark in a solution containing 0.2% DAB (Sigma-Aldrich) and 0.02% H_2_O_2_ in 50 mM Tris-HCl (pH 7.6) and washed another 5 times using cacodylate buffer. The samples were then embedded in Epon812 for sectioning.

### Plasmid construction and plant transformation

To specifically decrease the ROS level in pollen, *Arabidopsis CAT3* was amplified using the CAT3LP and CAT3RP primers and cloned into the pROKII vector to create the *Lat52*::*CAT3* construct for pollen-specific expression. This construct was transformed into wild-type Col-0 *Arabidopsis* plants by agrobacterium-mediated transformation. The amiRNA construct, which targets *KIN10*,*11*, was constructed as previously described [40]. The amiRNA precursor was amplified by overlapping PCR from the pRS300 template using the ImiR-s, IImiR-a, IIImiR*-s, IVmiR*-a primers designed by WMD3 Web microRNA Designer (http://wmd3.weigelworld.org/cgi-bin/webapp.cgi) (S3 Table). The sequenced fragment containing the amiRNA foldback in the cloning vector was subsequently subcloned into the pROKII vector under the control of Lat52 promoter to form LAT52::*amiRNA-KIN10*,*11* for pollen-specific expression inhibition. This construct was transformed into the wild-type Col-0 *Arabidopsis* plants by Agrobacterium-mediated transformation. All of the sequences of primers are listed in S3 Table.

### ROS staining for pollen grains

To label ROS, pollen grains were incubated in 10 μM CM-H2DCFDA (Cat# C6827; Molecular Probes) dissolved in 20 mM HEPES buffer (pH 7.2) for 5–10 min at room temperature. After washing with HEPES buffer, the pollen grains were observed under a LEICA TCS SP5 II LSCM (Leica, Wetzlar, Germany) using a 488-nm laser as the excitation light, and the emissions were collected at 505–530 nm. To analyze the relative ROS level in the pollen labeled by CM-H2DCFDA, photos were taken under the same LSCM microscopic settings. The relative fluorescence intensities of the pollen grains indicate the relative ROS levels analyzed by ImageJ software (http://rsb.info.nih.gov).

### Quantitative real-time-PCR (qRT-PCR) analysis

The mature pollen grains of wild-type plants, *kinβγ-1/+* and *kinβγ-2/+* mutant, Lat52::amiRNA-*KIN10*,*11* and Lat52::*CAT3* transgenic lines were collected. Total RNAs of pollen grains were extracted according to the manufacture using Ultrapure RNA Kit (TIANGEN, China) and cDNAs were got by reverse transcription using PrimerScript RT reagent Kit with gDNA Eraser-Perfect Real Time (TAKARA, Dalian). qRT-PCR reaction was performed with BIO-RAD CFX96 real-time system using iQ SYBR Green Supermix (BIO-RAD, Singapore) using the corresponding primer pairs (S3 Table). The RNA levels were normalized to that of TUBULIN2 with three biological replicates and three technical replicates.

## Supporting Information

S1 Fig Analysis of *kinβγ* T-DNA insertion lines. (A) Schematic representation of the *KINβγ* gene and the positions of T-DNA insertion in *kinβγ-1/+* and *kinβγ-2/+*. (B) Identification of *kinβγ-1/+* and *kinβγ-2/+* mutants using a PCR-based method with gene-specific primers. (C) The relative expression levels of *KINβγ* in pollen of the wild type, *kinβγ-1/+* and *kinβγ-2/+* determined by qRT-PCR analysis. The expression level in the wild type was set to 1.0. The error bars represent the SD of three biological replicates.
(DOC)

S2 Fig Pollen grain morphology of the *SNF4-YFP/+* and *SNF4-YFP/+ kinβγ-1/-*. (A) Pollen grains of the *SNF4-YFP/+* observed by SEM. (B) Pollen grains of *SNF4-YFP/+ kinβγ-1/-* observed by SEM. Arrow indicates the pollen grain with sunken surface. (C) Statistical analysis of abnormal pollen with sunken surfaces in the *SNF4-YFP/+* and *SNF4-YFP/+ kinβγ-1/-*. Data were collected from three independent experiments. No significant difference was detected (Student’s *t*-test, P = 0.464). Bars, 50 μm.
(DOC)

S3 Fig Ultrastructure of both the coats and walls of mature pollen. No significant difference in appearance was detected between the coat and wall of the wild type (A) and the mutants, *kinβγ-1/+* (B) and *kinβγ-2/+ qrt1*/- (C). Ba, bacula; In, intine; Ne, nexine; PC, pollen coat; Tc, tectum. Bars, 5 μm.
(DOC)

S4 Fig Pollen phenotypes of Lat52::amiRNA*-KIN10*,*11* transgenic lines. (A) Alexander staining to detect the viability of mature pollen. (B) Pollen morphology under SEM. Arrows indicate the pollen grains with abnormal morphology. (C) Mitochondria in pollen stained with MitoTracker Deep Red. (D) The relative expression levels of *KIN10* and *KIN11* in the wild type and the four transgenic lines determined by qRT-PCR analysis. The expression level in the wild type was set to 1.0. The error bars represent the SD of three biological replicates. Bars, 50 μm in (A), and 20 μm in (B) and (C).
(DOC)

S5 Fig Alexander staining and relative expression levels of *CAT3* in *Lat52*::*CAT3* transgenic lines. (A–E) Alexander staining of mature pollen of the wild-type (A) and transgenic lines overexpressing *Arabidopsis CAT3*, including line 1 (B), line 5 (C), line 13 (D), and line 14 (E). (F) qRT-PCR analysis of the relative expression levels of *CAT3* in the wild type and the four transgenic lines. The expression level in the wild type was set to 1.0. The error bars represent the SD of three biological replicates. (G, H) Alexander staining of mature pollen of two transgenic lines, line 3 (G), and line 9 (H), showing the shrunken and nonviable pollen grains. Bars, 25 μm in (A–E) and 200 μm in (G, H).
(DOC)

S6 Fig Analysis of pollen diameter in medium containing different concentrations of PEG4000 and H_2_O_2_. (A) Percentages of the pollen (short diameter < 17 μm) of the wild type and the *kinβγ-2/+* mutant in the medium containing 35% and 55% PEG4000, respectively. (B) Percentages of the *kinβγ-1/+* pollen (short diameter > 17 μm) in the medium containing 55% PEG4000 and H_2_O_2_. The error bars represent the SD of three biological replicates.
(DOC)

S1 Table Segregation analysis of selfed progenies of *kinβγ*/+ mutants. (DOC)

S2 Table Analysis of the genetic transmission efficiency (TE) of kinβγ alleles. (DOC)

S3 Table Primers used in this study. (DOC)
