# Supplementary material for: The Arabidopsis KINβγ Subunit of the SnRK1 Complex Regulates Pollen Hydration on the Stigma by Mediating the Level of Reactive Oxygen Species in Pollen
Source: PLoS Genet. 2016 Jul 29;12(7):e1006228. doi: 10.1371/journal.pgen.1006228 (PMC4966946; doi:10.1371/journal.pgen.1006228)
Supplement: S1 Fig — (A) Schematic representation of the KINβγ gene and the positions of T-DNA insertion in kinβγ-1/+ and kinβγ-2/+. (B) Identification of kinβγ-1/+ and kinβγ-2/+ mutants using a PCR-based method with gene-specific primers. (C) The relative expression levels of KINβγ in pollen of the wild type, kinβγ-1/+ and kinβγ-2/+ determined by qRT-PCR analysis. The expression level in the wild type was set to 1.0. The error bars represent the SD of three biological replicates. (DOC) [file pgen.1006228.s001.doc]

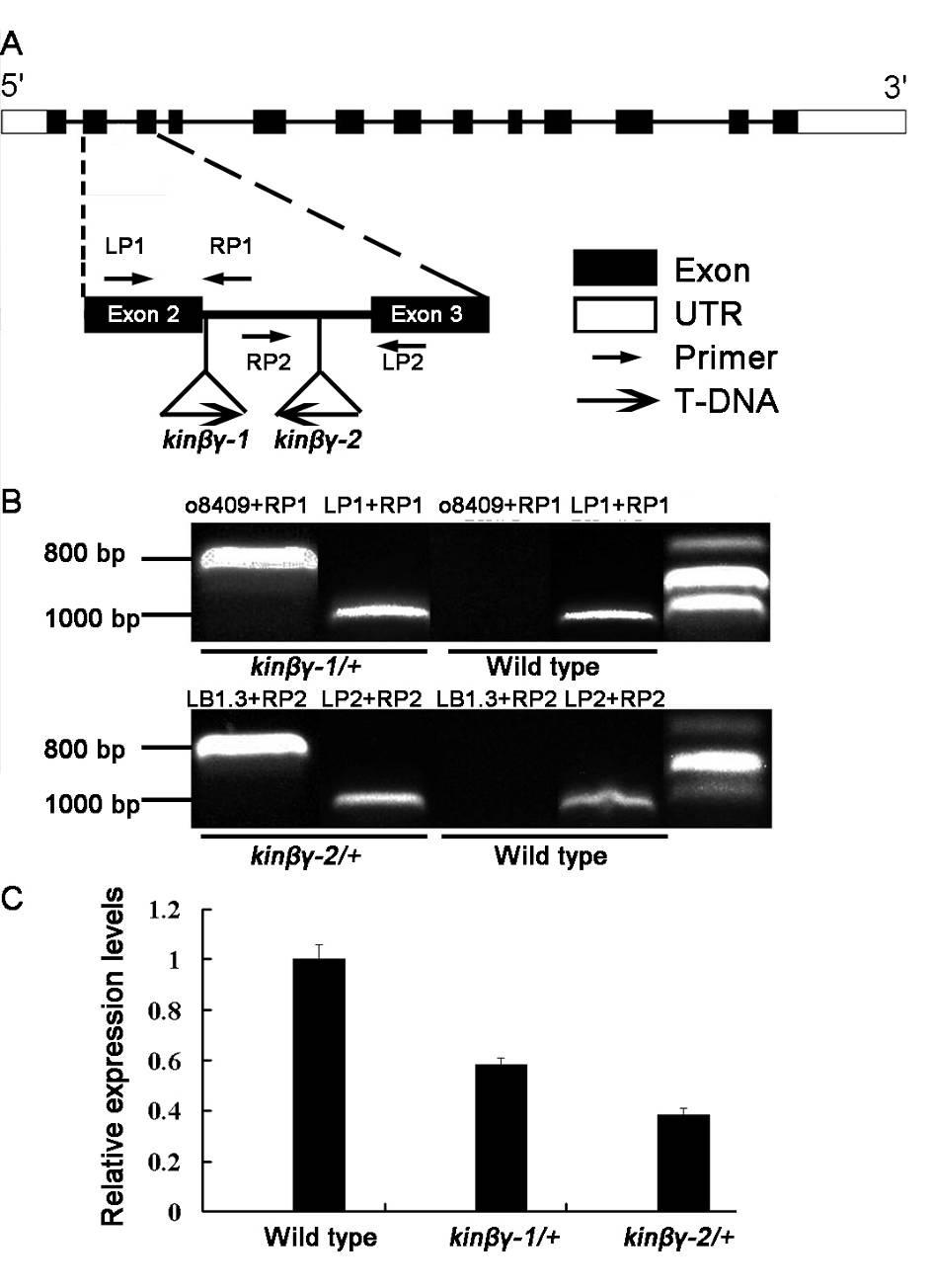


**S1 Fig. Analysis of *kinβγ* T-DNA insertion lines.**

(A) Schematic representation of the *KINβγ* gene and the positions of T-DNA insertion in *kinβγ-1/+* and *kinβγ-2/+*. (B) Identification of *kinβγ-1/+* and *kinβγ-2/+* mutants using a PCR-based method with gene-specific primers. (C) The relative expression levels of *KINβγ* in pollen of the wild type, *kinβγ-1/+* and *kinβγ-2/+* determined by qRT-PCR analysis. The expression level in the wild type was set to 1.0. The error bars represent the SD of three biological replicates.
