# Supplementary material for: The Arabidopsis KINβγ Subunit of the SnRK1 Complex Regulates Pollen Hydration on the Stigma by Mediating the Level of Reactive Oxygen Species in Pollen
Source: PLoS Genet. 2016 Jul 29;12(7):e1006228. doi: 10.1371/journal.pgen.1006228 (PMC4966946; doi:10.1371/journal.pgen.1006228)
Supplement: S2 Fig — (A) Pollen grains of the SNF4-YFP/+ observed by SEM. (B) Pollen grains of SNF4-YFP/+ kinβγ-1/- observed by SEM. Arrow indicates the pollen grain with sunken surface. (C) Statistical analysis of abnormal pollen with sunken surfaces in the SNF4-YFP/+ and SNF4-YFP/+ kinβγ-1/-. Data were collected from three independent experiments. No significant difference was detected (Student’s t-test, P = 0.464). Bars, 50 μm. (DOC) [file pgen.1006228.s002.doc]

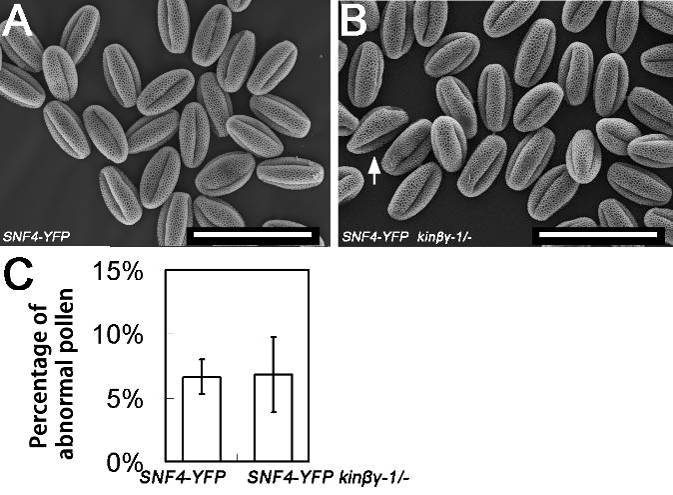


**S2 Fig. Pollen grain morphology of the *SNF4-YFP/+* and *SNF4-YFP/+ kinβγ-1/-*.**

(A) Pollen grains of the *SNF4-YFP/+* observed by SEM. (B) Pollen grains of *SNF4-YFP/+ kinβγ-1/-* observed by SEM. Arrow indicates the pollen grain with sunken surface. (C) Statistical analysis of abnormal pollen with sunken surfaces in the *SNF4-YFP/+* and *SNF4-YFP/+ kinβγ-1/-.* Data were collected from three independent experiments. No significant difference was detected (Student’s *t*-test, P=0.464). Bars, 50 µm.
