# Supplementary material for: The Arabidopsis KINβγ Subunit of the SnRK1 Complex Regulates Pollen Hydration on the Stigma by Mediating the Level of Reactive Oxygen Species in Pollen
Source: PLoS Genet. 2016 Jul 29;12(7):e1006228. doi: 10.1371/journal.pgen.1006228 (PMC4966946; doi:10.1371/journal.pgen.1006228)
Supplement: S3 Fig — No significant difference in appearance was detected between the coat and wall of the wild type (A) and the mutants, kinβγ-1/+ (B) and kinβγ-2/+ qrt1/- (C). Ba, bacula; In, intine; Ne, nexine; PC, pollen coat; Tc, tectum. Bars, 5 μm. (DOC) [file pgen.1006228.s003.doc]

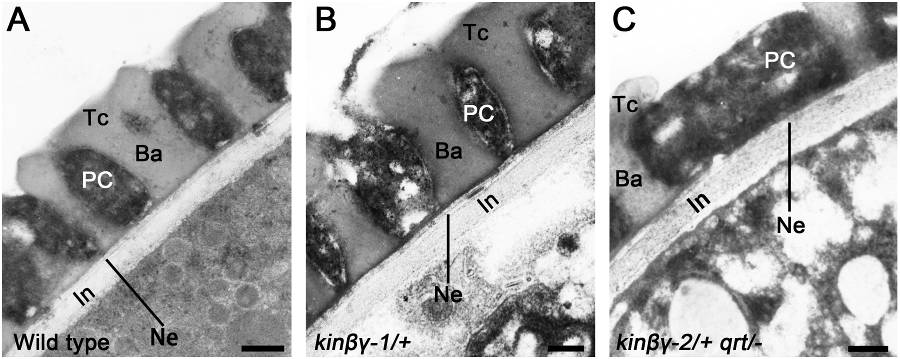


**S3 Fig. Ultrastructure of both the coats and walls of mature pollen.**

No significant difference in appearance was detected between the coat and wall of the wild type (A) and the mutants, *kinβγ-1/+* (B) and *kinβγ-2/+* *qrt1*/- (C). Ba, bacula; In, intine; Ne, nexine; PC, pollen coat; Tc, tectum. Bars, 5 µm.
