# Supplementary material for: The Arabidopsis KINβγ Subunit of the SnRK1 Complex Regulates Pollen Hydration on the Stigma by Mediating the Level of Reactive Oxygen Species in Pollen
Source: PLoS Genet. 2016 Jul 29;12(7):e1006228. doi: 10.1371/journal.pgen.1006228 (PMC4966946; doi:10.1371/journal.pgen.1006228)
Supplement: S4 Fig — (A) Alexander staining to detect the viability of mature pollen. (B) Pollen morphology under SEM. Arrows indicate the pollen grains with abnormal morphology. (C) Mitochondria in pollen stained with MitoTracker Deep Red. (D) The relative expression levels of KIN10 and KIN11 in the wild type and the four transgenic lines determined by qRT-PCR analysis. The expression level in the wild type was set to 1.0. The error bars represent the SD of three biological replicates. Bars, 50 μm in (A), and 20 μm in (B) and (C). (DOC) [file pgen.1006228.s004.doc]

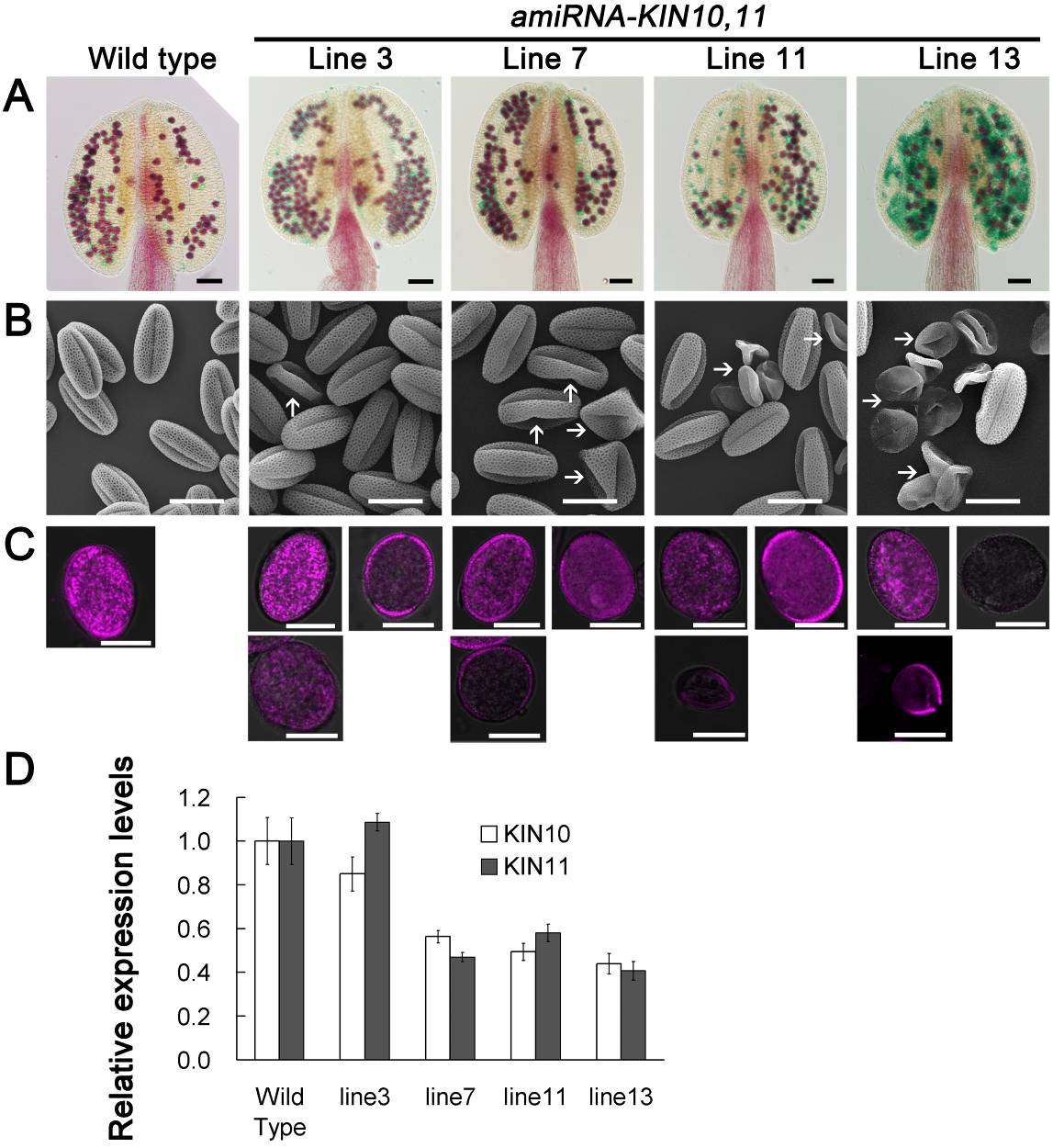


**S4 Fig. Pollen phenotypes of Lat52*::*amiRNA*-KIN10,11* transgenic lines.**

(A) Alexander staining to detect the viability of mature pollen. (B) Pollen morphology under SEM. Arrows indicate the pollen grains with abnormal morphology. (C) Mitochondria in pollen stained with MitoTracker Deep Red. (D) The relative expression levels of *KIN10* and *KIN11* in the wild type and the four transgenic lines determined by qRT-PCR analysis. The expression level in the wild type was set to 1.0. The error bars represent the SD of three biological replicates. Bars, 50 µm in (A), and 20 µm in (B) and (C).
