# Supplementary material for: The Arabidopsis KINβγ Subunit of the SnRK1 Complex Regulates Pollen Hydration on the Stigma by Mediating the Level of Reactive Oxygen Species in Pollen
Source: PLoS Genet. 2016 Jul 29;12(7):e1006228. doi: 10.1371/journal.pgen.1006228 (PMC4966946; doi:10.1371/journal.pgen.1006228)
Supplement: S5 Fig — (A–E) Alexander staining of mature pollen of the wild-type (A) and transgenic lines overexpressing Arabidopsis CAT3, including line 1 (B), line 5 (C), line 13 (D), and line 14 (E). (F) qRT-PCR analysis of the relative expression levels of CAT3 in the wild type and the four transgenic lines. The expression level in the wild type was set to 1.0. The error bars represent the SD of three biological replicates. (G, H) Alexander staining of mature pollen of two transgenic lines, line 3 (G), and line 9 (H), showing the shrunken and nonviable pollen grains. Bars, 25 μm in (A–E) and 200 μm in (G, H). (DOC) [file pgen.1006228.s005.doc]

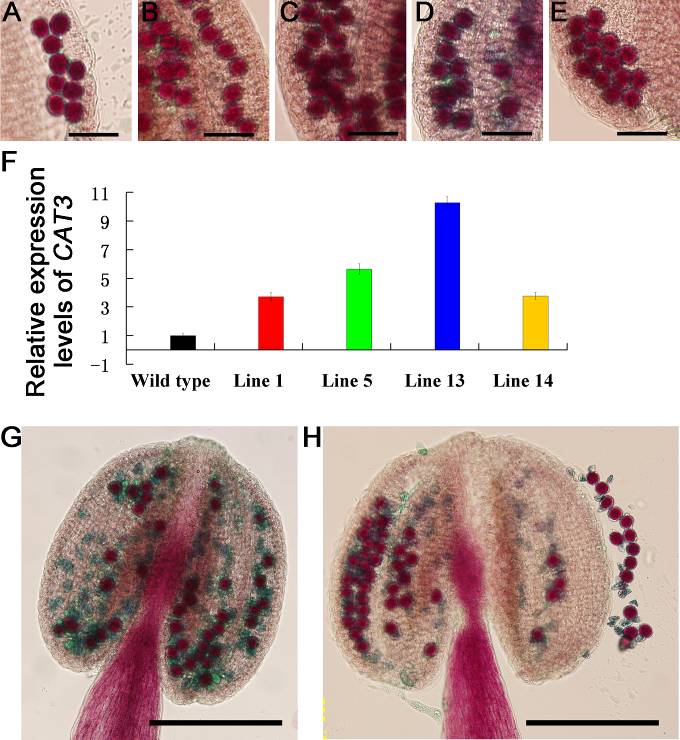


**S5 Fig. Alexander staining and relative expression levels of *CAT3* in *Lat52*::*CAT3* transgenic lines*.***

(A–E) Alexander staining of mature pollen of the wild-type (A) and transgenic lines overexpressing *Arabidopsis* *CAT3*, including line 1 (B), line 5 (C), line 13 (D), and line 14 (E). (F) qRT-PCR analysis of the relative expression levels of *CAT3* in the wild type and the four transgenic lines. The expression level in the wild type was set to 1.0. The error bars represent the SD of three biological replicates. (G, H) Alexander staining of mature pollen of two transgenic lines, line 3 (G), and line 9 (H), showing some shrunken and nonviable pollen grains. Bars, 25 µm in (A–E) and 200 µm in (G, H).
