# Supplementary material for: The Arabidopsis KINβγ Subunit of the SnRK1 Complex Regulates Pollen Hydration on the Stigma by Mediating the Level of Reactive Oxygen Species in Pollen
Source: PLoS Genet. 2016 Jul 29;12(7):e1006228. doi: 10.1371/journal.pgen.1006228 (PMC4966946; doi:10.1371/journal.pgen.1006228)
Supplement: S6 Fig — (A) Percentages of the pollen (short diameter < 17 μm) of the wild type and the kinβγ-2/+ mutant in the medium containing 35% and 55% PEG4000, respectively. (B) Percentages of the kinβγ-1/+ pollen (short diameter > 17 μm) in the medium containing 55% PEG4000 and H2O2. The error bars represent the SD of three biological replicates. (DOC) [file pgen.1006228.s006.doc]

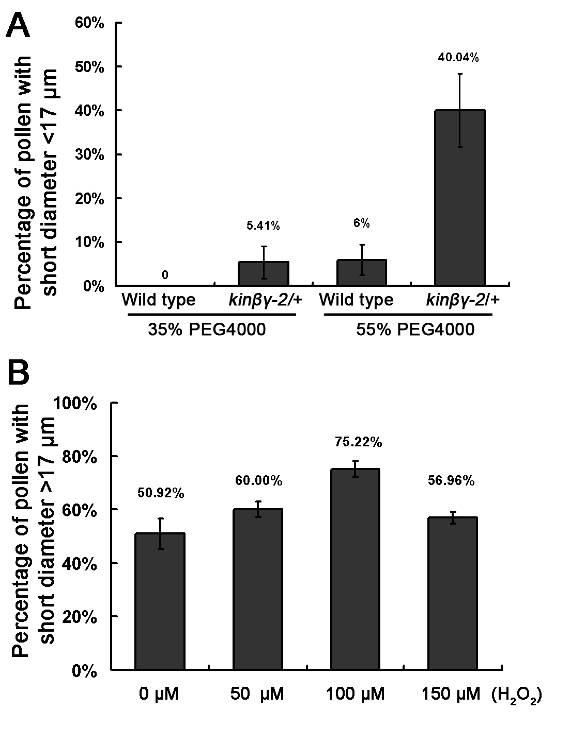


**S6 Fig. Analysis of pollen diameter in medium containing different concentrations of PEG4000 and H_2_O_2_.**

(A) Percentages of the pollen (short diameter < 17 µm) of the wild type and the *kinβγ-2/+* mutant in the medium containing 35% and 55% PEG4000, respectively.

(B) Percentages of the *kinβγ-1/+* pollen (short diameter > 17 µm) in the medium containing 55% PEG4000 and H_2_O_2_.

The error bars represent the SD of three biological replicates.
