# Supplementary material for: The Arabidopsis KINβγ Subunit of the SnRK1 Complex Regulates Pollen Hydration on the Stigma by Mediating the Level of Reactive Oxygen Species in Pollen
Source: PLoS Genet. 2016 Jul 29;12(7):e1006228. doi: 10.1371/journal.pgen.1006228 (PMC4966946; doi:10.1371/journal.pgen.1006228)
Supplement: S1 Table — (DOC) [file pgen.1006228.s007.doc]

**S1 Table Segregation analysis of selfed progenies of *kinβγ***/+ mutants

|  | *KINβγ*/+* | *kinβγ*/+ | *kinβγ*/- | Ratio |
| --- | --- | --- | --- | --- |
| *kinβγ-1*/+ | 115 | 93 | 0 | 1: 0.81: 0 |
| *kinβγ-2*/+ | 138 | 133 | 0 | 1: 0.96: 0 |
| * The genotypes of progenies were analyzed by a PCR-based method as described in the “Materials and Methods.” | | | | |
