# Supplementary material for: The Arabidopsis KINβγ Subunit of the SnRK1 Complex Regulates Pollen Hydration on the Stigma by Mediating the Level of Reactive Oxygen Species in Pollen
Source: PLoS Genet. 2016 Jul 29;12(7):e1006228. doi: 10.1371/journal.pgen.1006228 (PMC4966946; doi:10.1371/journal.pgen.1006228)
Supplement: S2 Table — (DOC) [file pgen.1006228.s008.doc]

**S2 Table Analysis of the genetic transmission efficiency (TE) of *kinβγ*** alleles

| ♀ × ♂ | *KINβγ*/+ | *kinβγ*/+ | TE (%)^#^ |
| --- | --- | --- | --- |
| *kinβγ-1/+* × Wild type | 79 | 78 | 98.7* |
| Wild type × *kinβγ-1/+* | 220 | 6 | 2.7* |
| *kinβγ-2/+* × Wild type | 127 | 98 | 77.2^Δ^ |
| Wild type × *kinβγ-2/+* | 165 | 12 | 7.3^Δ^ |
| ^#^ The TEs of *kinβγ* alleles = *kinβγ*/+ / *KINβγ/+* × 100%.  * and ^Δ^ indicating there are significant differences of transmission efficiency between them (Student’s *t* test, *P* < 0.01). | | | |
