# Supplementary material for: The Arabidopsis KINβγ Subunit of the SnRK1 Complex Regulates Pollen Hydration on the Stigma by Mediating the Level of Reactive Oxygen Species in Pollen
Source: PLoS Genet. 2016 Jul 29;12(7):e1006228. doi: 10.1371/journal.pgen.1006228 (PMC4966946; doi:10.1371/journal.pgen.1006228)
Supplement: S3 Table — (DOC) [file pgen.1006228.s009.doc]

**S3 Table Primers used in this study.**

| **Primer** | **Sequence (5'—3')** |
| --- | --- |
| *kinβγ-1* LP (for mutant) | TTCGTGTTGGTGGAATTTCTC |
| *kinβγ-1* RP (for mutant) | CACCATTTCCGCTTACAAATG |
| *kinβγ-2* LP (for mutant) | GAGAAAGCTCCAAATCAACCC |
| *kinβγ-2* RP (for mutant) | CTCAGGGCAGCTTCTTACTCC |
| LBb1.3 (for mutant) | ATTTTGCCGATTTCGGAAC |
| o8409 (for mutant) | ATATTGACCATCATACTCATTGC |
|  |  |
| *KINβγ* LP (qRT-PCR) | TGGCAGCTGTTCCGGTTATA |
| *KINβγ* RP (qRT-PCR) | CTCCGATTCTAGGGACCCAC |
|  |  |
| *CAT3* LP (*Lat52::CAT3*) | AACTCGAGATGGATCCTTACAAGTATCGTCCT |
| *CAT3* RP *(Lat52::CAT3)* | TTGGTACCGATGCTTGGCCTCACGTTCAGA |
|  |  |
| *KIN10,11 I miR-s* (*Lat52::amiRNA-KIN10,11*) | gaTAAAAAATGACCATCTGGCATtctctcttttgtattcc |
| *KIN10,11 II miR-a* (*Lat52::amiRNA-KIN10,11*) | gaATGCCAGATGGTCATTTTTTAtcaaagagaatcaatga |
| *KIN10,11 III miR*s*  (*Lat52::amiRNA-KIN10,11*) | gaATACCAGATGGTCTTTTTTTTtcacaggtcgtgatatg |
| *KIN10,11 IV miR*a*  (*Lat52::amiRNA-KIN10,11*) | gaAAAAAAAAGACCATCTGGTATtctacatatatattcct |
|  |  |
| *CAT3* LP (qRT-PCR) | AGGTCCGGTTCTTCTTGAGG |
| *CAT3* RP (qRT-PCR) | GTGGACAACGGTGGAGAAAC |
